# A signalling cascade involving receptor-activated phospholipase A_2_, glycerophosphoinositol 4-phosphate, Shp1 and Src in the activation of cell motility

**DOI:** 10.1186/s12964-019-0329-3

**Published:** 2019-03-01

**Authors:** Alessia Varone, Stefania Mariggiò, Manpreet Patheja, Vincenzo Maione, Antonio Varriale, Mariangela Vessichelli, Daniela Spano, Fabio Formiggini, Matteo Lo Monte, Nadia Brancati, Maria Frucci, Pompea Del Vecchio, Sabato D’Auria, Angela Flagiello, Clara Iannuzzi, Alberto Luini, Piero Pucci, Lucia Banci, Carmen Valente, Daniela Corda

**Affiliations:** 1 Institute of Protein Biochemistry, National Research Council, Via Pietro Castellino 111, 80131 Naples, Italy; 2 Magnetic Resonance Centre (CERM), University of Florence, 50019 Sesto Fiorentino, Italy; 3 Institute of Food Science, National Research Council, Via Roma 64, 83100 Avellino, Italy; 4 Italian Institute of Technology, Centre for Advanced Biomaterials for Health Care at CRIB, Largo Barsanti e Matteucci 53, 80125 Naples, Italy; 5 Institute of High Performance Computing and Networking, National Research Council, Via P. Castellino 111, 80131 Naples, Italy; 6 Department of Chemical Sciences, University of Naples Federico II, Via Cintia, 80126 Naples, Italy; 7 CEINGE Advanced Biotechnology, Via G. Salvatore 486, 80145 Naples, Italy; 8 Department of Biochemistry, Biophysics and General Pathology, Second University of Naples, Via L. de Crecchio 7, 80138 Naples, Italy

**Keywords:** Shp1, EGF, SH2 domain, Glycerophosphoinositols, Phosphoinositides, Actin polymerisation, Membrane ruffles, Cell motility

## Abstract

**Background:**

Shp1, a tyrosine-phosphatase-1 containing the Src-homology 2 (SH2) domain, is involved in inflammatory and immune reactions, where it regulates diverse signalling pathways, usually by limiting cell responses through dephosphorylation of target molecules. Moreover, Shp1 regulates actin dynamics. One Shp1 target is Src, which controls many cellular functions including actin dynamics. Src has been previously shown to be activated by a signalling cascade initiated by the cytosolic-phospholipase A_2_ (cPLA_2_) metabolite glycerophosphoinositol 4-phosphate (GroPIns4*P*), which enhances actin polymerisation and motility. While the signalling cascade downstream Src has been fully defined, the mechanism by which GroPIns4*P* activates Src remains unknown.

**Methods:**

Affinity chromatography, mass spectrometry and co-immunoprecipitation studies were employed to identify the GroPIns4*P-*interactors; among these Shp1 was selected for further analysis. The specific Shp1 residues interacting with GroPIns4*P* were revealed by NMR and validated by site-directed mutagenesis and biophysical methods such as circular dichroism, isothermal calorimetry, fluorescence spectroscopy, surface plasmon resonance and computational modelling. Morphological and motility assays were performed in NIH3T3 fibroblasts.

**Results:**

We find that Shp1 is the direct cellular target of GroPIns4*P*. GroPIns4*P* directly binds to the Shp1-SH2 domain region (with the crucial residues being Ser 118, Arg 138 and Ser 140) and thereby promotes the association between Shp1 and Src, and the dephosphorylation of the Src-inhibitory phosphotyrosine in position 530, resulting in Src activation. As a consequence, fibroblast cells exposed to GroPIns4*P* show significantly enhanced wound healing capability, indicating that GroPIns4*P* has a stimulatory role to activate fibroblast migration. GroPIns4*P* is produced by cPLA_2_ upon stimulation by diverse receptors, including the EGF receptor. Indeed, endogenously-produced GroPIns4*P* was shown to mediate the EGF-induced cell motility.

**Conclusions:**

This study identifies a so-far undescribed mechanism of Shp1/Src modulation that promotes cell motility and that is dependent on the cPLA_2_ metabolite GroPIns4*P*. We show that GroPIns4*P* is required for EGF-induced fibroblast migration and that it is part of a cPLA_2_/GroPIns4*P/*Shp1/Src cascade that might have broad implications for studies of immune-inflammatory response and cancer.

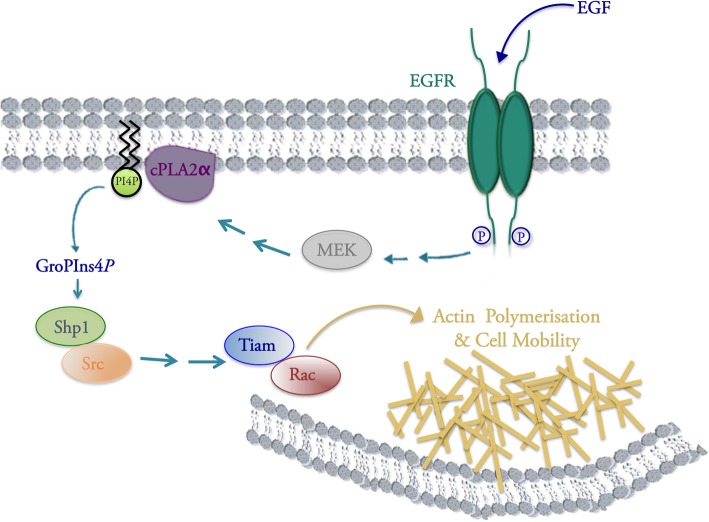

**Electronic supplementary material:**

The online version of this article (10.1186/s12964-019-0329-3) contains supplementary material, which is available to authorized users.

## Background

Glycerophosphoinositols are ubiquitous cellular metabolites derived from the deacylation of the membrane phosphoinositides by receptor-activated cytosolic phospholipase A_2_α (cPLA_2_α) [1, 2]. Receptors activating the cPLA_2_α/glycerophosphoinositol pathway include adrenergic, purinergic and tyrosine kinase receptors such as epidermal growth factor (EGF) or insulin [3–5]. Also lysophosphatidic-acid receptor in fibroblasts [6], Fc receptor in macrophages [7], lipopolysaccharide-TLR4 in human monocytes [8] lead to glycerophosphoinositol production by activating cPLA_2_α. The glycerophosphoinositol formation is particularly abundant in hematopoietic cells where these compounds are involved in multiple signalling pathways in the context of inflammation and immune reactions [9], generally exerting inhibitory effects [7, 8]. In addition, a major effect of glycerophosphoinositol 4-phosphate (GroPIns4*P*; the phosphorylated form of glycerophosphoinositol, GroPIns) is the regulation of the actin cytoskeleton dynamics in fibroblasts and immune cells [10–12].

We have previously characterised in depth the activation of the actin cytoskeleton by GroPIns4*P*, and reported that this metabolite induces membrane ruffling by triggering a phosphorylation cascade initiated by the Src kinase (and, possibly, by the two isoforms of Src) [11]. In NIH3T3 fibroblasts, exogenously added GroPIns4*P*, acting through Src, led to the phosphorylation – and hence activation – of PLCγ, thereby causing an increase in intracellular Ca^2+^ levels and the activation of calcium-calmodulin kinase II (CaMKII). In turn, CaMKII induced the activation of the GTPase Rac1 at the plasma membrane by engaging its GTP exchange factor (GEF), Tiam1, finally leading to ruffle formation [11]. A similar pathway controlling actin dynamics was observed in Jurkat T-cells, in which exogenously-added GroPIns4*P* promotes actin polymerisation by activating the Lck kinase, and inducing the phosphorylation of the GDP/GTP exchanger Vav and subsequent activation of the GTPase Rac, resulting in increased cell motility [9, 12]. This GroPIns4*P*-dependent Lck activation potentiates the transactivation of the TCR by the SDF1α-stimulated CXCR4 receptor thus enhancing T-cell chemotaxic response [12]. These effects of GroPIns4*P* might play a role in the immune response by mediating the recruitment of T-cells toward the injured site [9, 12].

Despite the many studies on the numerous and important biological activities of the glycerophosphoinositols [13, 14], the molecular target/s of these metabolites have not yet been identified leaving a major gap in our understanding of their cellular activities.

In this study, we have attempted the isolation of the direct interactors/receptors of the glycerophosphoinositols by pull-down assay coupled with liquid chromatography-tandem mass-spectrometry analysis. Among the molecules identified, we focused on the protein tyrosine-phosphatase 1 (Shp1) because of its well-known role in Src activation and cytoskeleton organisation [15, 16]. Shp1 is a member of the SH2-domain-containing family of non-membrane protein-tyrosine phosphatases expressed in most cells but particularly abundant in hematopoietic cells [17, 18]. It has been implicated in the negative regulation of various receptor-mediated pathways such as the cytokine and chemokine-receptors, T- and B-cell receptors as well as growth factor receptors [15, 16]. Mice deficient in Shp1 (*motheaten* or *viable motheaten*) are affected by several immunological, inflammatory, and haematological abnormalities [19, 20]. Studies of hematopoietic cells from these mice indicate that Shp1 plays also an important role in the regulation of the SDF1α-induced signalling pathway [21]. In addition, Shp1 has been shown to have a positive role in regulating the actin cytoskeleton and in activating the Src kinase [15, 22]. Here Shp1 acts by interacting with the Src phosphotyrosine residues and dephosphorylates the inhibitory Tyr530 leading to Src activation [15].

These effects appear to be strikingly reminiscent of those reported for the GroPIns4*P*-dependent signalling cascade leading to the control of the actin cytoskeleton [10–12]. Prompted by these similarities we examined the interaction between GroPIns4*P* and Shp1, focussing in particular on the Src activation of the actin cytoskeleton dynamics. We find that GroPIns4*P* binds to Shp1, through its C-terminal SH2 domain. This binding then leads to enhanced interaction between Shp1 and Src and to Shp1-dependent dephosphorylation and activation of Src kinase which, in turn, results in the induction of actin-dependent ruffling and increased fibroblast cell motility. As these effects are part of the motogenic, pro-invasion activity typically induced by growth factor receptors, we examined whether the GroPIns4*P*/Shp1 cascade might be required for such activity. We report evidence indicating that this is indeed the case.

These observations represent a breakthrough in our understanding of the physio-pathological roles of both the glycerophosphoinositols and of Shp1. They indicate that these molecules play a role in receptor-activated pathways that lead to the activation of PLA_2_, followed by generation of GroPIns4*P* and the activation of Shp1, with important consequences on cell motility. Given the potent activation of PLA_2_ in several cells involved in the primary immune response, the GroPIns4*P*/Shp1 cascade is likely to play a role also in inflammatory reactions.

## Methods

### Antibodies, cDNAs and reagents

NIH3T3 and Raw264.7 cells were obtained from the American Type Culture Collection (ATCC, USA). Dulbecco’s Modified Eagle’s Medium (DMEM), OptiMEM, foetal bovine serum (FBS), calf serum (CS), penicillin, streptomycin, trypsin-EDTA, L-glutamine and the Lipofectamine LTX/plus reagent were obtained from Gibco BRL (Grand Island, NY, USA). Wild-type Shp1 and Shp1-C455S were kindly provided by Dr. Axel Ullrich (Max Planck Institute of Biochemistry, Martinsried, Germany). Src construct was kindly provided by S. Gutkind (NIH, Bethesda, MD, USA).

For the antibodies: anti-p-Src (phosphorylated on tyrosine 530, p-Tyr530), anti-p-Src (phosphorylated on tyrosine 418, p-Tyr418), anti-c-Src, anti-p-PLCγ1 (phosphorylated on tyrosine 783, p-Tyr783) and anti-PLCγ1 antibodies were obtained from Cell Signaling Technology, Inc. (Beverly, MA, USA); the rabbit polyclonal anti-Shp1 and goat polyclonal anti-cPLA_2_α were obtained from Santa Cruz (Santa Cruz, CA, USA); and the mouse monoclonal anti-Src [clone 327] was obtained from Abcam (Cambridge, UK). Alexa Fluor 488-phalloidin and the Alexa 488- and Alexa 546-conjugated goat anti-rabbit and anti-mouse antibodies were obtained from Molecular Probes (Oregon, USA).

GroPIns4*P* and GroPIns4*P*-Bio were obtained from Echelon Biosciences (Salt Lake City, UT, USA). Inositol 4-phosphate (Ins4*P*) was obtained from Cayman chemicals (Ann Arbor, MI, USA). EGF and PDGF were obtained from Sigma-Aldrich (St. Louis, MO, USA). Protein tyrosine-phosphatase (PTPase) inhibitor I (TPI-1), Shp1/2 PTPase inhibitor (NSC-87877), cPLA_2_α inhibitor (pyrrolidine) and goat polyclonal HRP-conjugated anti-rabbit and anti-mouse antibodies were obtained from Calbiochem (CA, USA). Dynabeads MyOne Streptavidin C1 beads were obtained from Invitrogen/Gibco. Protein-A Sepharose beads, Protein-G Sepharose beads and Glutathione Sepharose 4B matrix were obtained from Amersham Pharmacia Biotech (NJ, USA). Benzamidine Sepharose 4 Fast Flow and the sensor Chip SA were obtained from GE Healthcare (Germany). All other reagents were of the highest purities from standard commercial sources.

### Plasmid construction

To generate the CFP-Shp1 construct, PCR primers were designed to add an EcoRI site to the 5′-end and a XhoI site to the 3′-end of the human Shp1 variant 2 sequence. The PCR product was cut with EcoRI and XhoI and inserted in the correct reading frame into pECFP-C3 (BD Biosciences) cut with the same two enzymes. The same procedure was used to clone Src cDNA into the pEYFP-N1 vector (BD Biosciences). To generate the Shp1-Flag construct, PCR primers were designed to add an EcoRI site to the 5′-end and a XbaI site to the 3′-end of the human Shp1 variant 2 sequence. The PCR product was cut with EcoRI and XbaI and cloned into p3X Flag CMV-14 (Sigma) cut with the same enzymes. The Shp1-GFP construct was generated by cloning the human Shp1 variant 2 sequence, amplified by PCR, into the pEGFP-N3 vector (BD Biosciences).

The primers were designed to add an EcoRI site to the 5′-end and a SacII site, preceded by A to keep Shp1 in the correct frame, to the 3′-end of Shp1. The 6xHis-tagged fusion proteins used in this study were derived by sub-cloning the following cDNA- or PCR-amplified fragments into pETM11 containing a multiple cloning site inserted into the NcoI/NotI sites: the C-terminal truncated form of human Shp1 cDNA (His-Shp1; amino acids 1–529), Shp1 NH2- and COOH-terminal SH2 domains [His-SH2 (N + C); amino acids 1–221] and Shp1 catalytic domain (His-PTPase; amino acids 245–529). The Shp1 S118A/R138E/S140A triple mutant was generated by two sequential site-directed mutagenesis reactions. The primers used for the mutagenesis reaction to generate the Shp1 R138E/S140A mutant were 5′-GGACGTTTCTTGTGGAGGAGGCCCTCAGCCAGCCTGG-3′ and 5′-CCAGGCTGGCTGAGGGCCTCCTCCACAAGAAACGTCC-3′. The primers used for the mutagenesis reaction to generate the Shp1 S118A mutant were 5′-GGTACCATGGCCACATGGCTGGCGGGCAGGCAG-3′ and 5′-CTGCCTGCCCGCCAGCCATGTGGCCATGGTACC-3′. All recombinant plasmids were verified by DNA sequencing.

### Cell culture, transfection and treatments

NIH3T3 and Raw 264.7 cells were cultured in DMEM supplemented with 2 mM L-glutamine, 50 U/mL penicillin, 50 mg/mL streptomycin, and 10% CS or heat-inactivated FBS (30 min at 55 °C), respectively. NIH3T3 cells were transfected with different plasmids using the Lipofectamine LTX/plus reagent according to the manufacturer’s instructions. For immunofluorescence experiments, NIH3T3 cells were seeded onto glass coverslips in 24-well plates at a concentration suitable for 70% confluence without transfection or 50% confluence in the case of Lipofectamine-based cell transfection. In the latter, approximately 24 h after seeding, NIH3T3 cells were transiently transfected with different plasmids with the Lipofectamine LTX/plus reagent following the manufacturer’s instructions. Before treatments, NIH3T3 cells were serum-starved in DMEM with 2 mM glutamine, 50 U/mL penicillin and streptomycin for 16–20 h. The cells were then treated with different stimuli and/or inhibitors, as indicated in the text and/or Figure legends. Addition of exogenous GroPIns4*P* was at 50 μM (unless otherwise indicated), a concentration eliciting an intracellular concentration of about 1.5 μM, as calculated from the Nernst equation (with T = 300 K, z = − 3, and Veq = − 30 mV, an average value for cultured, non-excitable cells).

### GroPIns4*P*-bio pull-down assay

Raw 264.7 cells maintained in the appropriate growth medium were detached using PBS-EDTA, centrifuged at 300×*g*, washed three times with PBS and re-suspended in lysis buffer (20 mM Tris, pH 7.4, 150 mM KCl, 5 mM MgCl_2_, 1 mM DTT, 5 mM EGTA, 1% Triton X-100) supplemented with a protease inhibitor cocktail (Complete Mini EDTA-free, Roche). The cell lysate was kept on a rotating wheel for 30 min at 4 °C. The lysate was then centrifuged at 13,000×*g* for 10 min. The supernatant obtained from the centrifugation was recovered, brought to a 0.2% (*w*/*v*) final concentration in Triton X-100, and dialysed twice against 1000 volumes PBS at 4 °C. The dialysis step was performed to remove endogenous GroPIns4*P*. Ten milligrams of cell extract for each sample was pre-cleared on 1 mg of uncoupled streptavidin-conjugated paramagnetic beads for 4 h at 4 °C on a rotating wheel.

Later, the lysates were recovered and incubated for 16 h at 4 °C with 1 mg of streptavidin-conjugated beads that were previously incubated with 2.5 nmoles of GroPIns4*P*-Bio or biotin in binding buffer (50 mM Tris-HCl, pH 7.6, 50 mM KCl, 10 mM EDTA) supplemented with the protease inhibitor cocktail. Following incubation, the unbound materials were separated by a magnetic particle concentrator, and the beads were washed five times with 2 ml of binding buffer. GroPIns4*P*-bound proteins were specifically eluted with 5 mM GroPIns4*P*, which corresponded to a 100-fold excess compared to the number of moles of GroPIns4*P* immobilised on the beads. The elution was performed for 30 min at 4 °C on a rotating wheel. Following the incubation, the proteins eluted by GroPIns4*P* were recovered using a magnetic particle concentrator, and the beads were re-suspended in 100 μL of SDS sample buffer. Both fractions were eluted by specific displacement, and the SDS sample buffer was analysed by 10% SDS/PAGE. The gel was then stained with GelCode Blue Stain Reagent (according to the manufacturer’s instructions). The bands were analysed by LC-MS/MS. For GroPIns4*P*-Bio pull-down assays with purified Shp1, 1 μg of purified Shp1 was incubated for 16 h at 4 °C with 1 mg of streptavidin-conjugated paramagnetic beads in the presence of 2.5 nmoles of biotin or GroPIns4*P*-Bio in binding buffer plus protease inhibitors. Following this incubation, the unbound material and beads were washed with binding buffer. The beads with bound protein were boiled in 100 μL of SDS sample buffer, separated by 10% SDS-PAGE, and transferred onto nitrocellulose membranes for western blotting.

### Expression and purification of recombinant proteins

pETM11 constructs encoding His-tagged Shp1 fragments were transformed into *E. coli* BL21(DE3) bacteria. The transformed bacteria were grown to an OD_600_ of 0.6, and expression of recombinant proteins was induced by the addition of IPTG (0.1 mM). After overnight incubation at 20 °C, the cells were harvested by centrifugation at 6000 rpm for 10 min and rinsed twice with PBS. The pellet was re-suspended in lysis buffer (25 mM Tris-HCl, pH 7.5, 150 mM NaCl, 10 mM β- mercaptoethanol, 20 mM imidazole) containing protease inhibitor cocktail as described above, and lysozyme, MgCl_2_ and DNase I were added at final concentrations of 0.5 mg/mL, 5 mM and 0.1 mg/mL, respectively. The suspension was incubated at 4 °C for 30 min and sonicated on ice 8 times for 15 s. Subsequently Triton X-100 was added to a final concentration of 1% *w*/*v*, and the mixture was incubated for 15 min at 4 °C. The bacterial lysate was then centrifuged at 22,000 rpm for 30 min at 4 °C. The supernatant was applied to a Ni-NTA-agarose column that had been previously equilibrated in lysis buffer for 3 h at 4 °C. After elution with lysis buffer containing 250 mM imidazole, His-Shp1 was dialysed twice against 1000 volumes of PBS and stored in aliquots at − 80 °C.

### Fluorescence spectroscopy

The absorption spectra of Shp1 were recorded using a Varian Cary 1 spectrophotometer. Steady-state fluorescence emission measurements were performed with a K2 fluorometer (ISS, Champaign, IL, USA) equipped with a two-cell temperature-controlled sample holder. To selectively excite Trp residues, the excitation wavelength was set at 295 nm with a slit width of 1 nm. To avoid the inner-filter effect, all steady-state fluorescence measurements were performed on Shp1 with optical densities at 295 nm less than 0.1 OD. All measurements were made at 25 °C with the temperature of the samples determined in the cuvette with an accuracy of ±0.2 °C. For each experiment, the Shp1 concentration was fixed at 1.2 μM, while GroPIns4*P* was varied from 0.0 to 5.0 μM. Thus, a 500 μL solution containing 1.2 μM Shp1 dissolved in PBS pH 7.4 was titrated by successive additions of GroPIns4*P* (dissolved in PBS buffer, pH 7.4). After each addition, the samples were incubated for 2 min and fluorescence emission spectra were recorded between 310 and 410 nm in triplicate. To exclude the influence of sample dilution, fluorescence titration experiments were performed by adding the same amount of PBS buffer in the total volume (500 μL).

### Surface plasmon resonance

The instrument used for surface plasmon resonance was a BIAcore 2000 (BIAcore AB, Uppsala, Sweden). Carboxymethylated dextran pre-immobilised with streptavidin chip (SA chip) was first cleaned with three consecutive 1-min injections of a solution of 1 M NaCl in 50 mM NaOH before the immobilisation procedure. Five minutes after the cleaning process, when the sensorgram reached a stable baseline, GroPIns4*P*-Bio was diluted in running buffer (HBS-EP buffer: 10 mM Hepes, 150 mM NaCl, 5 mM EDTA 0.005% Surfactant P20, pH 7.4) (GL/min. To obtain the maximum immobilisation level of GroPIns4*P*-Bio on the surface, multiple injections were performed.

Following GroPIns4*P*-Bio immobilisation, SPR binding analysis was performed in the concentration range of 0.0 to 5.0 μM of Shp1. The Shp1 stock solution was diluted in 100 μL of HBS-EP buffer at the specific concentrations. The binding flow was fixed to 20 μL/min, and the time of injection was 3 min. After each injection of protein, the binding surface was washed with a buffer containing 2.0 mM glycine/HCl, pH 3.0. A blank flow cell was used as the reference surface to eliminate the bulk effect. Data management and calculation of the kinetics parameters were performed using the BIAevaluation 3.1 software (BIAcore AB, Uppsala, Sweden).

For the competition experiments, SPR assays were performed at a fixed concentration of Shp1 (1.2 μM) in the presence of increasing concentrations of GroPIns4*P* (0.0 to 5.0 μM). Both Shp1 and GroPIns4*P* were dissolved in HBS-EP buffer and incubated for 10 min before injection onto the SA sensor chip. The flow was fixed to 20 μL/min, and the time of injection was 3 min. A solution of 2.0 mM glycine/HCl, pH 3.0, was used in the regeneration step to remove the analyte bond before reusing the sensor chip.

### Isothermal titration calorimetry (ITC)

ITC measurements were performed by using a Nano-ITC III (TA instruments, New Castle, DE, USA) with a reaction cell volume of 1 ml kept at 25 °C. A protein solution of 10 μM was titrated into the calorimetric cell with a solution of 50–100 μM of the ligand GroPIns4*P* (Ins4*P*) in the syringe. A sequence of 16 injections of 15 μL volume was programmed with a stirring speed of 250 rpm at 500 s intervals. Protein samples were prepared with protein solutions at 25 °C in 20 mM Tris buffer pH 7.4 with 50 mM NaCl and 1 mM TCEP. To minimise the differences in buffer composition and pH between cell and syringe, both ligand and protein solutions were prepared with the same batch of buffer. The heat of dilution of the GroPIns4*P* into the buffer solution was measured in a separate experiment and appropriate corrections were made. The heat evolved after each ligand injection was obtained from the integral of the calorimetric signal. Raw data were integrated, corrected for non-specific heats and normalized for concentration. The equilibrium dissociation constant (K_D_), binding enthalpy (ΔbH) and binding stoichiometry (n) were obtained by nonlinear regression of the experimental data using a model of independent binding sites, by means of the NanoAnalyze software. Experiments were performed in duplicate.

### Nuclear magnetic resonance spectroscopy

A suite of triple resonance experiments (Additional file 1 Table S1) was used for assignment of the ^1^H, ^15^N and ^13^C backbone resonances of the Shp1 cSH2 domain. All experiments were acquired on a 700 MHz Bruker Avance instrument equipped with a TCI cryoprobe. Assignment of the cSH2 domain backbone resonances was complete with the exception of the resonances of S_109_ and G_175_. ^15^N cSH2 (200 μM) was titrated with GroPIns4*P*, and a series of ^1^H–^15^N-HSQC spectra were recorded at different cSH2:GroPIns4*P* ratios up to a 1:5 ratio. Distinct chemical shift changes were observed in the ^1^H–^15^N-HSQC spectra upon addition of GroPIns4*P*, according to a fast exchange regime. The ^1^HN and ^15^N chemical shift perturbations were estimated by using the following equation [23]:

$$ \varDelta \updelta ={\left[\left(\varDelta {\mathrm{HN}}^2+{\left(\varDelta \mathrm{N}/5\right)}^2\right)/2\right]}^{1/2} $$

Where ΔHN and ΔN are the differences between the ^1^H and ^15^N chemical shifts of free cSH2 and cSH2 in the presence of GroPIns4*P*, respectively.

### Docking calculations

Docking calculations were performed with HADDOCK2.2 implemented in the WeNMR/West-Life GRID-enabled web portal (www.wenmr.eu). The docking calculations were driven by ambiguous interaction restraints between all solvent-exposed residues involved in the intermolecular interactions [24]. The docking calculations were performed using the cSH2 domain (stretch 110–213) of the crystal structure of human tyrosine phosphatase Shp1 (PDB code: 2B3O) [25]. The active residues of the Shp1 cSH2 domain were defined as residues those showing a chemical shift perturbation upon GroPIns4*P* binding and with at least 50% solvent accessibility. The residues S_118_, G_120_, E_139_, S_140_ and S_142_ were defined as active. Passive residues were defined as residues close in space to the active residues and with at least 50% solvent accessibility. The residue solvent accessibility was calculated with the program NACCESS. In the initial rigid body docking calculation phase, 5000 structures of the complex were generated, and the best 400 in terms of total intermolecular energy were further submitted to further calculations and to a final refinement in water. The final 400 structures were then clustered using a cutoff of 5.0 Å of the Root-Mean-Square Deviation (RMSD). Sixteen clusters were obtained and ranked according to their HADDOCK score. Among them, 5 clusters exhibited acceptable values in terms of their energetic and scoring functions (i.e. having an HADDOCK score lower than − 66) (Additional file 1 Table S2).

### Immunofluorescence analysis for membrane ruffling

For ruffle assessment after treatments, immunofluorescence labelling and morphological analysis of the cells were performed as previously described in a double-blind fashion [11]. In brief, the assessment was based on a null score (zero) for the absence of the feature, followed by a score of one or two according to the level of response of each individual cell, where one indicate that ruffling was confined to one area of the cell (< 25% of the cell circumference), and two indicate that two or more discrete areas of the cell contained ruffles. The cell phenotypes were quantified by counting 150 cells in each sample. The data were then expressed as percentage scores (±SD) for each treatment response compared with its respective control. Scoring of the control cells was performed in the same way to evaluate the presence of the features of interest in untreated cells.

### Wound-healing assay

To perform the wound-healing assay, NIH3T3 cells were seeded in 6-well tissue culture plates to a final density of 3 × 10^5^ cells/well and maintained at 37 °C and 5% CO_2_ for 24 h until confluence before being starved overnight in DMEM containing 1% bovine serum. The cell monolayer was wounded by scratching with a 10 μL standard sterile pipette tip. The scratched monolayer was rinsed twice with DMEM containing 1% bovine serum to remove cell debris and incubated with or without 50 μM GroPIns4*P*. Phase contrast images were taken after 24 h, and the size of the wound area was determined automatically using image-processing techniques for an accurate, unbiased process. In detail, the input image was processed by applying a Sobel edge detector [26], obtaining a gradient image. A threshold process [27] was then performed on the gradient image to obtain the foreground corresponding to the wound area. Particularly, all of the pixels of the gradient image with values less than the threshold *θ* were assumed to be pixels of the foreground (experimentally, *θ* is set to 4). Finally, procedures based on morphological operations were used to remove small regions and to fill holes of the wound area to complete detection and analysis [28].

### Western blotting and immunoprecipitation

NIH3T3 cells were washed with ice-cold PBS and lysed on ice in buffer containing 20 mM Tris-HCl, pH 8.0, 150 mM NaCl, 1% Triton-X100, 5 mM Na_3_VO_4_, 30 mM β-glycerophosphate and 10 mM NaF supplemented with the protease inhibitor cocktail and processed for SDS-PAGE. Westen blotting was performed with the relevant antibodies. For immunoprecipitation experiments, 2 μg of an anti-Shp1 antibody or 5 μg of an anti-Src antibody were added to 1 mg of NIH3T3 cell lysates at 4 °C and incubated overnight. The samples were then incubated with A or G protein agarose beads for 1 h at 4 °C. Western blotting was performed from the washed and denatured complexes. The extent of Src co-immunoprecipitated was evaluated using an NIH imaging system and normalised for the amount of total Shp1 immunoprecipitated.

### In-vitro dephosphorylation of Src

Src was immunoprecipitated from NIH3T3 cells as described above. The immunoprecipitates were washed three times with lysis buffer, three times with lysis buffer without phosphatase inhibitors, and twice with phosphatase buffer (100 mM Na-Hepes, pH 7.4, 150 mM NaCl, 1 mM EDTA, and 10 mM DTT). The immunoprecipitates were then split equally into different tubes for treatment with Shp1 or buffer only. The treatment consisted of the addition of 2 μg of Shp1 to the immunoprecipitates in the absence or presence of 50 μM GroPIns4*P* and an incubation at 37 °C for different times. The reactions were terminated by the addition of concentrated SDS-sample buffer. The samples were then resolved by SDS-PAGE, and the level of Tyr530 phosphorylation was determined by western blotting and normalised for the total amount of immunoprecipitated Src.

### Acceptor photobleaching measurements

Acceptor photobleaching (apFRET) experiments were carried out with Leica SP5 confocal microscope, as follows: for CFP (donor) fluorescence detection, excitation at 458 nm and fluorescence detected at 470–490 nm. For YFP bleaching, a 514 nm argon laser was used. apFRET was measured by increase in CFP fluorescence intensity before (IDA) and after (ID) YFP photobleaching. To ensure reproducibility and reliability of CFP fluorescence measurements in the absence of acceptor (ID), YFP was photobleached to 10% of its initial fluorescence. The FRET efficiency was calculated as E = (ID − IDA)/ ID .

## Results

### Identification of Shp1 as a GroPIns4*P*-interacting protein

To identify protein interactors of GroPIns4*P* we used a pull-down assay followed by liquid chromatography coupled to tandem mass spectrometry (LC/MS-MS) to identify the collected proteins. This procedure yielded 30 potential GroPIns4*P*-binding proteins (see Table S3 for full list and relative scores). Functional annotation of these proteins allowed their classification under five major clusters: cellular motility and membrane trafficking components; stress and folding proteins; metabolic enzymes; proteins associated with DNA and RNA processing; and cell-cycle regulation proteins.

We next searched these clusters for proteins that could be related to the activities of GroPIns4*P* on actin dynamics. The cellular motility and membrane trafficking cluster included the tyrosine-phosphatase Shp1, a known regulator of the Src kinase [15, 22]. GroPIns4*P* induces actin ruffle formation in fibroblasts through a signalling cascade that begins with Src activation; however, no direct interaction occurs between GroPIns4*P* and Src [11]. We thus hypothesised that Shp1 might represent the origin of this GroPIns4*P*- and Src-dependent pathway. Based on this hypothesis, we focussed on Shp1 and explored its possible role in GroPIns4*P*-induced activation of Src and actin cytoskeleton remodelling.

**Fig. 1:**
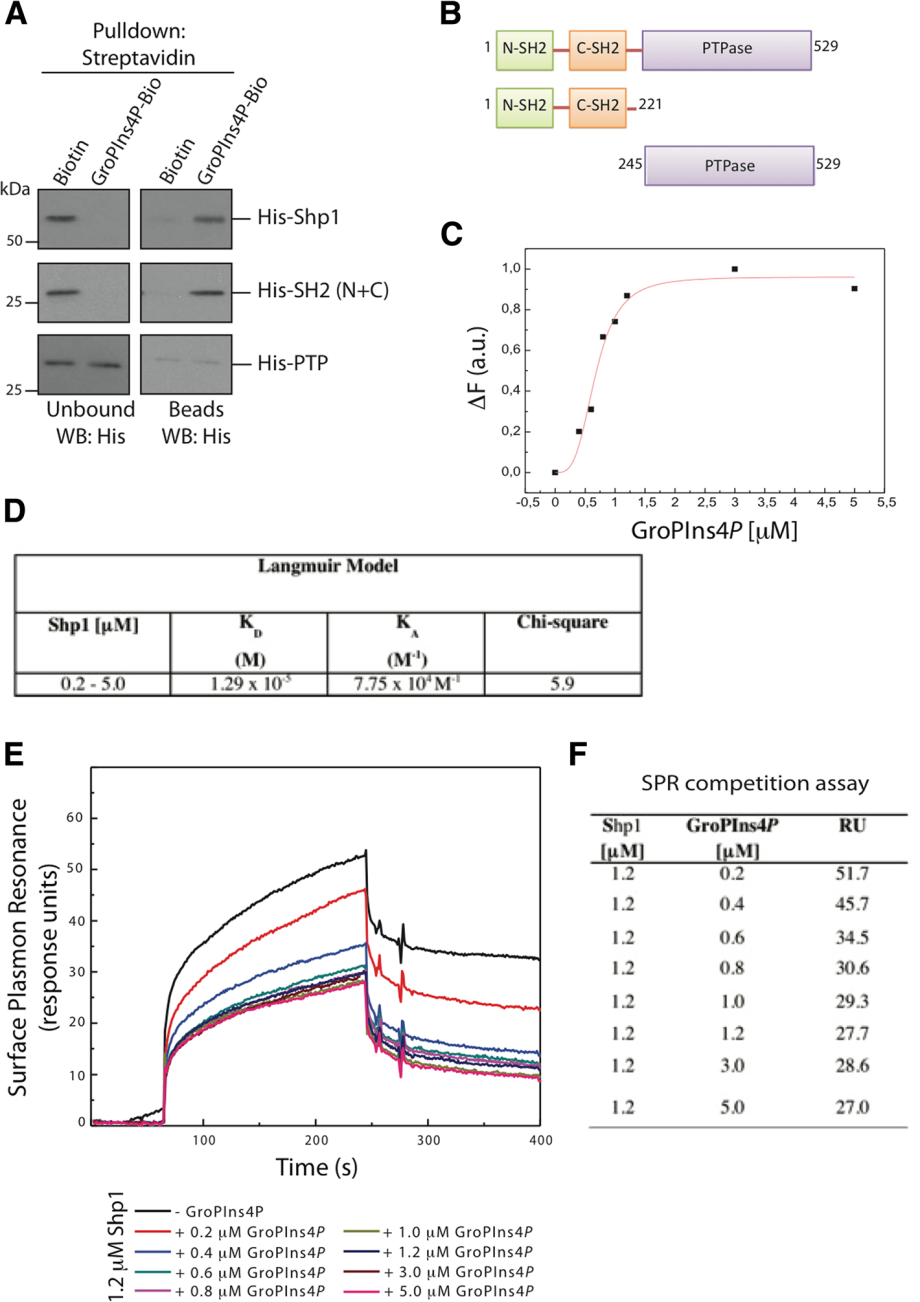
Direct binding of GroPIns4*P* to Shp1. **a** Representative pull-down of streptavidin-conjugated beads using Biotin or biotinylated GroPIns4*P* (GroPIns4*P*-Bio) with either Shp1_1–529_ (His-Shp1), the N-terminal SH2-domain mutant (His-SH2 (N + C)) or the catalytic-domain mutant (His-PTPase) of Shp1. Unbound and eluted (beads) proteins were analysed by western blotting using an anti-Shp1 antibody. Molecular weights (kDa) are indicated on the left of each panel. **b** Schematic domain structure illustrating the amino acid sequences of the Shp1 mutants used in the pull-down. **c** Dose-response effect of GroPIns4*P* on Shp1 fluorescence emission (ΔF) at 332 nm (fluorescence emission spectra of Shp1 upon addition of the indicated μM concentrations of GroPIns4*P* are shown in supplementary Additional file 1 Figure S1). **d** Kinetic interaction parameters calculated by surface plasmon resonance (SPR) analysis. Binding of Shp1 on a GroPIns4*P*-Bio-functionalised chip was analysed SPR as a function of time and analysed using the Langmuir fit. **e** SPR competition assay. Sensorgram showing Shp1 (1.2 μM) binding to immobilised GroPIns4*P*-Bio in the absence and presence of increasing concentrations of GroPIns4*P*. The GroPIns4*P* concentrations are indicated below the panel. **f** Data from SPR competition assay (RU = resonance units). Data are representative of three independent experiments, each performed in triplicate

We then asked whether Shp1 is a direct target of GroPIns4*P*, by in vitro pull-down assays using purified recombinant Shp1 with GroPIns4*P*-Bio-bound beads (Fig. 1a). Recombinant Shp1 was specifically pulled-down by GroPIns4*P*-Bio-bound beads (but not by control Biotin-bound beads), indicating that GroPIns4*P* binds directly to Shp1 (Fig. 1a).

### GroPIns4*P* directly binds to the SH2 domain of Shp1

In determining which region of Shp1 is involved in the interaction with GroPIns4*P*, we noted that Shp1 (which is composed of 597 amino acids) contains two SH2 domains connected by a short linker at the N-terminus, followed by a catalytic domain (the PTPase domain) and short C-terminal tail. The C-terminal tail is highly disordered and affects protein stability; thus, a tailless-form of Shp1 (residues 1–529) has previously been used [25] and it is used also in this study.

For our experiments, we constructed two His-tagged Shp1 truncated fragments: the N-terminal portion composed of the two SH2 domains [His-SH2 (N + C)], and the central catalytic domain (His-PTPase) (Fig. 1b). These purified recombinant Shp1 truncation mutants, as well as the Shp1 tailless 1–529 form, were used in pull-down assays with GroPIns4*P*-Bio. As shown in Fig. 1a, the His-SH2 (N + C) fragment fully retained the ability to bind to GroPIns4*P*-Bio, whereas the catalytic region of the enzyme (His-PTPase) was devoid of GroPIns4*P*-Bio-binding ability (Fig. 1a). These observations indicate that GroPIns4*P* binds to one or both SH2 domains of Shp1.

This direct interaction was further analysed by steady-state tryptophan (Trp) fluorescence emission spectroscopy using purified Shp1. We exploited the fact that there are nine Trp residues throughout Shp1, three of which fall in the His-SH2 (N + C) fragment. The addition of a ligand, GroPIns4*P* in this case, is expected to cause Trp fluorescence quenching if GroPIns4*P* binds sufficiently close to a Trp residue. When Shp1 was incubated with different concentrations of GroPIns4*P*, the Shp1 signals in the Trp emission spectra decreased as a function of the GroPIns4*P* concentration, with maximal quenching of the initial emission of 18% at 5 μM GroPIns4*P* and an apparent EC50 of approximately 0.6 μM (Fig. 1c and S1a). These results indicate direct saturable binding of GroPIns4*P* to Shp1. Notably, the concentrations used in our experiments (Fig. 1c) are comfortably within the range of the GroPIns4*P* levels evaluated in several cell types as in the low micromolar range [3, 5, 29].

By way of confirmation, surface plasmon resonance (SPR) was employed to measure the binding of increasing concentrations of Shp1 to GroPIns4*P*-Bio immobilised on a sensor chip. Shp1-GroPIns4*P* binding was clearly measurable and dose-dependent, with an apparent K_D_ of about 13 μM (Figs. 1d and S1b) [30]. This value appeared one order of magnitude higher than that obtained in the Trp-fluorescence quenching experiments, and it might be due to the different availability to binding of the immobilised and chemically-modified GroPIns4*P*-Bio vs the free compound.

To further analyse this aspect, we first determined the Shp1 concentration that was able to saturate the binding sites on the GroPIns4*P*-Bio-functionalised chip (1.2 μM under our experimental conditions). Then, to determine the binding affinity between GroPIns4*P* and Shp1, we performed SPR competitive-binding experiments using GroPIns4*P*-Bio immobilised on a sensor chip, the saturating concentration of Shp1 and increasing concentrations of non-biotinylated, “free” GroPIns4*P* to compete for binding to the sensor chip. Free-GroPIns4*P* competed with the binding of Shp1 to the GroPIns4*P*-Bio-functionalised chip with an EC50 of approximately 0.8 μM (Figs. 1e-f), in line with the results obtained with the Shp1 Trp fluorescence analysis (Fig. 1c).

Finally isothermal titration calorimetry (ITC) was employed to evaluate the binding of GroPIns4*P* to Shp1. The titration of Shp1 with GroPIns4*P* resulted in a binding stoichiometry of 1:1 and a K_D_ of 0.3 μM (Additional file 1 Figure S2). At the same time we evaluated by ITC also the binding of Ins4*P*, a degradation product of GroPIns4*P*; no binding was resolved for Ins4*P*, in line with observations we have previously reported indicating that Ins4*P* has no effect on actin cytoskeleton organisation under our experimental procedures [10].

Altogether, these results indicate that GroPIns4*P* binds Shp1 in its SH2-domain portion, and that the affinity of the binding is in the low μM range (0.3–0.8 μM), which is within the cellular physiological concentration range of this metabolite.

### Structural features of the GroPIns4*P* binding site in the C-terminal SH2 domain of Shp1

Since the portion of Shp1 necessary and sufficient for its interaction with GroPIns4*P* was mapped within the two SH2 domains, we further defined the interaction of this compound with Shp1 by nuclear magnetic resonance (NMR) spectroscopy. The purified C-terminal SH2 domain (cSH2) was selected for these studies, since its stability and solubility enable NMR characterisation in solution.

**Fig. 2:**
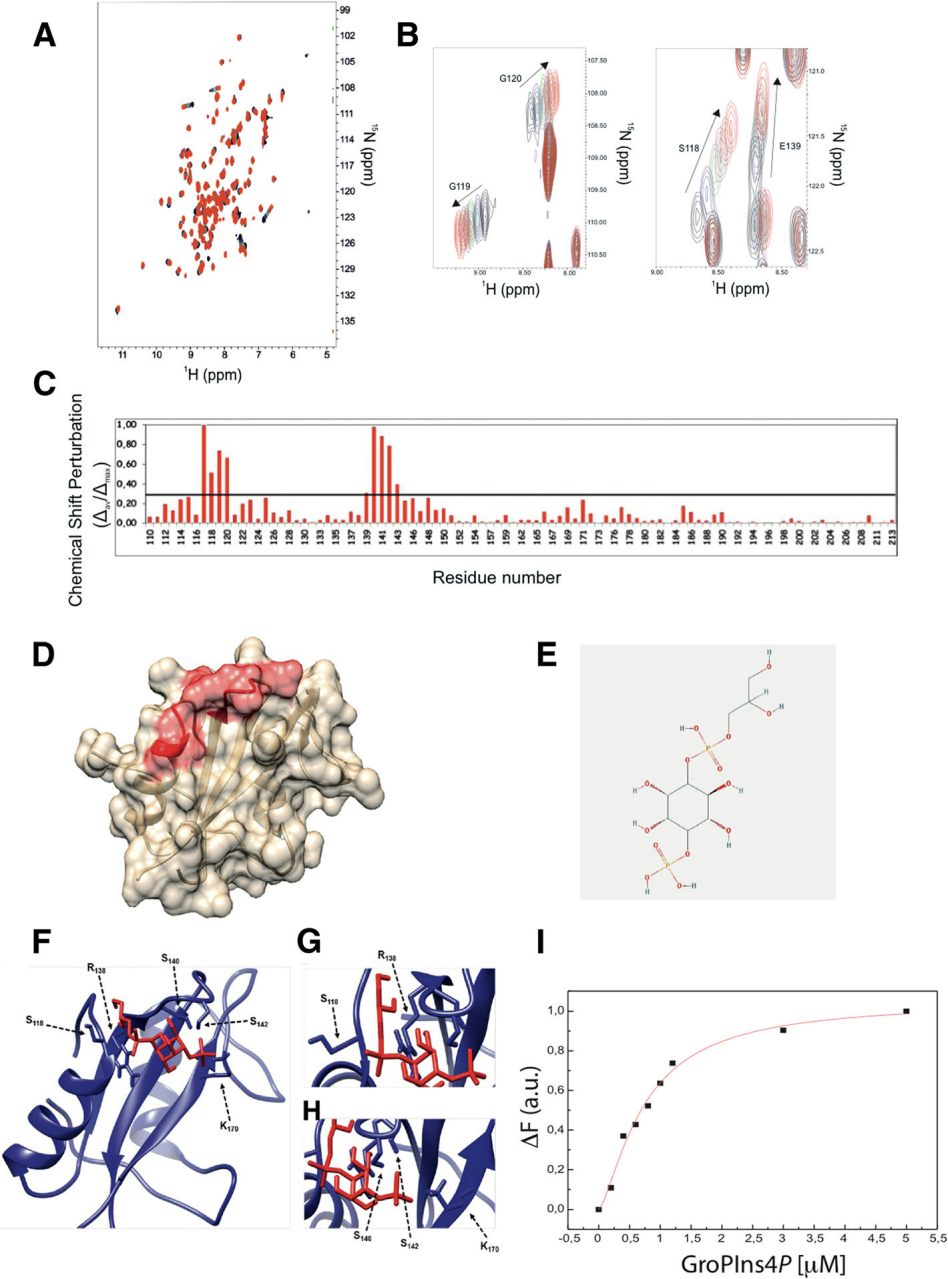
Identification of the Shp1-cSH2 domain residues involved in GroPIns4*P* binding. **a, b** Overlay of the ^1^H-^15^N Heteronuclear Single-Quantum Coherence (HSQC) spectra of the ^15^N-labeled cSH2 domain in the absence and presence of different amount of GroPIns4*P*. Protein alone (black) and in the presence of GroPIns4*P* at the ratios of 1:1 (blue), 1:2 (green), 1:3 (magenta), 1:4 (brown), 1:5 (red) are reported. **c** Normalised weighted average chemical shift differences (Δ_av_/Δ_max_) between the GroPIns4*P*-bound and free forms of the Shp1 cSH2 domain plotted against the residue number for the amide proton and nitrogen resonances. The horizontal bold line at 0.3 indicates the average value plus one standard deviation. **d** Chemical shift perturbation mapped onto the cSH2 structure of Shp1 (PDB code: 2B3O). The amino acid residues affected by GroPIns4*P* binding (Δ_av_/Δ_max_ ≥ 0.3) are depicted in red. **e** Chemical structure of GroPIns4*P.* **f** Ribbon representation of the complex, in which the cSH2 domain is in blue and GroPIns4*P* in red. Residues involved in the interaction are presented as sticks and are labelled. **g** Close view of S_118_ and R_138_ of the cSH2 domain that interact with the phosphate group of GroPIns4*P*. **h** Close view of S_140,_ S_142_ and K_170_ of the cSH2 domain that interact with the 4′-phosphate group of GroPIns4*P.* Similar experiments on the nSH2 domain of Shp1 were hampered by the poor stability of the isolated fragment. **i** Functional validation of the Shp1 S118A/R138E/S140A mutant. Dose-response effect of GroPIns4*P* on Shp1 S118A/R138E/S140A mutant fluorescence emission (ΔF) at 332 nm (fluorescence emission spectra of Shp1 upon addition of the indicated μM concentrations of GroPIns4*P* are shown in supplementary Additional file 1 Figure S3)

The ^1^H-^15^N HSQC spectrum acquired for the ^15^N-labelled cSH2 domain showed well-dispersed amide signals, typical of a folded protein [31]. Titration of cSH2 with GroPIns4*P*, up to a cSH2:GroPIns4*P* ratio of 1:5, induced chemical shift changes that increased with the GroPIns4*P* concentration, thus confirming the binding of GroPIns4*P* to this domain (Figs. 2a-b). This behaviour also indicated that the GroPIns4*P*-bound and free forms of the cSH2 domain exchange with one another at a rate faster than the resonance frequency differences in the two species. The exchange rate can be set at values higher than 2 × 10^3^ s^− 1^. The amino acid residues affected by GroPIns4*P* binding clustered in a well-defined region of the Shp1 protein (PDB code: 2B3O) [25] (Fig. 2b), which includes segments ^117^MSGG^120^ and ^139^ESLSQ^143^ (Figs. 2c-d) belonging to its pY-binding pocket [32]. This result is in line with previous studies suggesting that some SH2 domains bind lipids in their pY pockets [33, 34]. We next modelled the structure of the complex between the cSH2 domain and GroPIns4*P*, using HADDOCK2.2 docking calculations driven by chemical shift perturbation data. Five clusters of structural models exhibited acceptable values for the energetic and scoring functions (Additional file 1 Table S2). Comparison between the lowest energy structures of these clusters revealed that only cluster 2 showed an orientation of GroPIns4*P* bound to the Shp1 cSH2 domain (Figs. 2e-f) that was in full agreement with the experimental NMR data. In this structural model, the glycerol moiety of the GroPIns4*P* is embedded between the N-terminal region of Shp1 and the loop connecting its β1 and β2 strands, allowing the phosphate group of GroPIns4*P* to establish contacts with the side chain of Ser118 and Arg138 (β1) (Fig. 2g). Moreover, the inositol group is inserted in the pY-binding pocket, and the 4′ phosphate group of GroPIns4*P* interacts with the side chains of Ser140, Ser142 and Lys170 (β3) (Fig. 2h).

Based on this modelling, we produced a panel of proteins with mutations in the above amino acid residues, with the aim of investigating the relevance of the GroPIns4*P* binding site identified on Shp1-cSH2 in the context of the full-length protein. In each case, correct folding of the mutated proteins was verified by Circular Dichroism spectroscopy, and the mutated proteins were then assayed by Trp fluorescence emission spectroscopy for their ability to bind GroPIns4*P*. As predicted, the combination of S118A/R138E/S140A mutations significantly impaired GroPIns4*P* binding, as the binding efficiency of the mutated protein decreased by up to 50% (Figs. 2i and S3). Accordingly, the binding isotherm of Shp1 S118A/R138E/S140A mutant, obtained under the same conditions of the wild-type, was also reduced indicating a significantly lower affinity toward GroPIns4*P* (Additional file 1 Figure S2).

These data thus indicate that the Ser118, Arg138 and Ser140 residues contribute to the interaction of GroPIns4*P* with full-length Shp1.

### GroPIns4*P* induces Src activation in NIH3T3 cells

Next, we analysed the involvement of Shp1 in GroPIns4*P*-induced Src activation in NIH3T3 fibroblasts, the model system where the GroPIns4*P*/ Src-dependent formation of actin ruffles was first reported [11]. Src is regulated by opposing phosphorylation and dephosphorylation events. Phosphorylation by the C-terminal Src kinase (CSK) at Tyr530, promotes the intramolecular interaction of the C-terminal catalytic domain of Src with the SH2 and SH3 domains and blocks the kinase in an inactive conformation. Dephosphorylation of Tyr530 leads to the release of this block and the opening up of the molecule to assume an active state [22]. Shp1 has previously been shown to bind and dephosphorylate Src at Tyr530, thus inducing activation of the kinase [15].

**Fig. 3:**
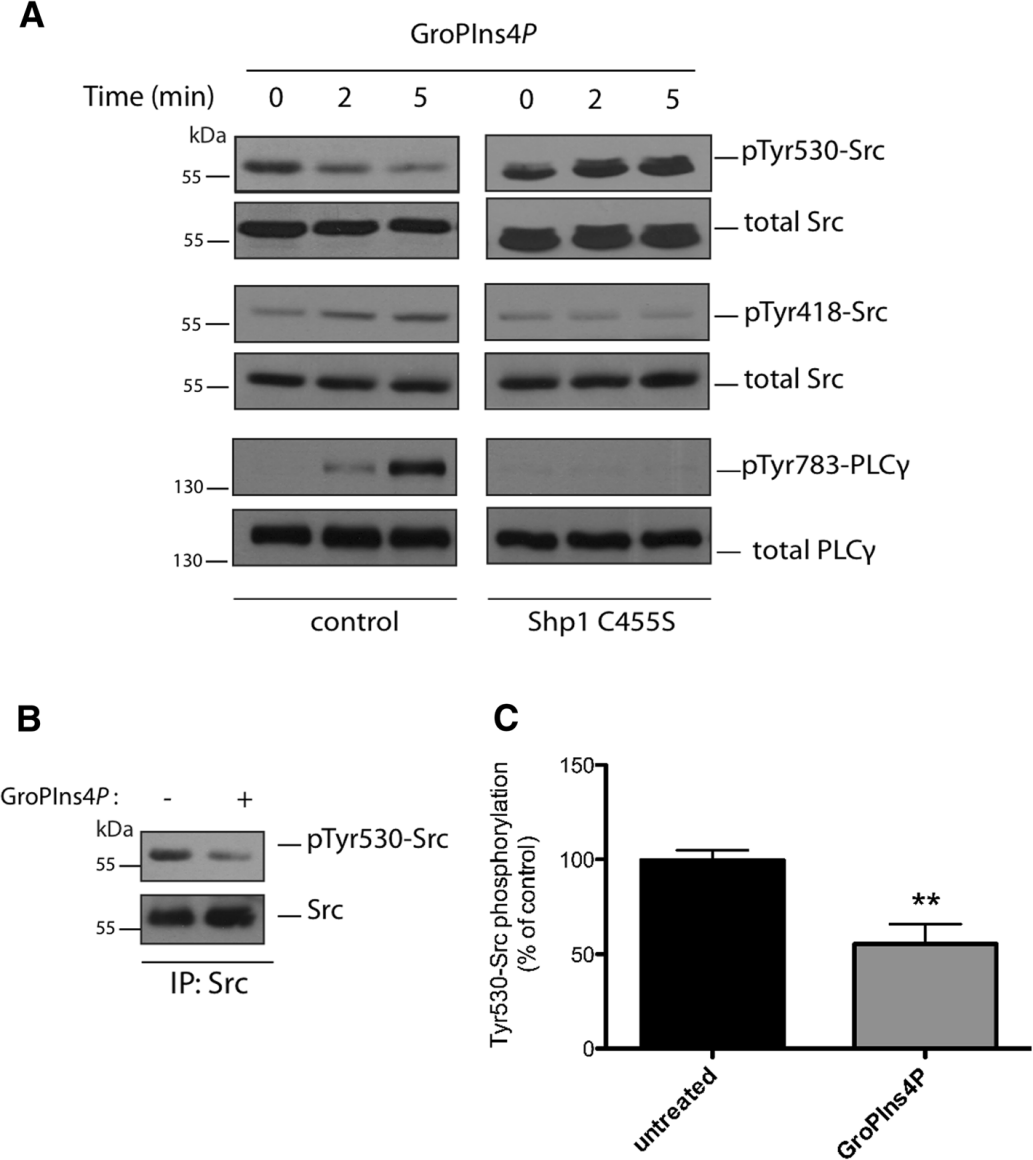
GroPIns4*P* induces Src dephosphorylation. **a** Representative western blots using anti-phosphotyrosine 530 in Src (pTyr530-Src), anti-phosphotyrosine 418 in Src (pTyr418-Src) and anti-phosphotyrosine 783 in PLCγ (pTyr783-PLCγ) specific antibodies in serum-starved NIH3T3 cells non-transfected or over-expressing the dominant negative Shp1-C455S mutant and treated with 50 μM GroPIns4*P* for the indicated times (see the top of the two panels). Total Src and total PLC were used as loading controls. Data are representative of three independent experiments. Molecular weight standards (kDa) are indicated on the left of each panel. **b** Representative immunoprecipitated Src fraction (IP: Src) from NIH3T3 cell lysates washed and incubated with purified recombinant Shp1 for 10 min at 37 °C in the absence (−) or presence (+) of 50 μM GroPIns4*P* (as indicated). The top panel shows western blots with an anti-phosphotyrosine antibody (pTyr530-Src) to reveal the specific phosphorylation of Tyr-530 in Src. The blot was then re-probed with an anti-Src antibody for immunoprecipitated proteins (bottom panel). Molecular weight standards (kDa) are indicated on the left of each panel. **c** Quantification of Src phosphorylation in samples treated with GroPIns4*P* (as in b) by the ImageJ analysis software. Data (GroPIns4*P*) are expressed as percentages of untreated sample (untreated) of the means (±SD) of three independent experiments, each of which was performed in duplicate (*n* = 6). **P < 0.02 (Student’s *t*-test)

We therefore examined whether this mechanism could account for GroPIns4*P*-induced Src activation in NIH3T3 cells. It should be noted here that although glycerophosphoinositols are hydrophilic molecules, they can cross the plasma membrane through specific permeases acting as bidirectional transporters in mammals [35], that are orthologues of the GIT1 transporter identified in yeast [36]. Cells were treated with 50 μM GroPIns4*P* for 2 to 5 min (see Methods for details) and lysates analysed using western blotting with an antibody that recognises the phosphorylated Tyr530 of Src. The Src inhibitory Tyr530 phosphorylation markedly decreased in response to treatment with GroPIns4*P* (Fig. 3a). Under these conditions, also the activating Tyr418 phosphorylation was increased and both modifications increased the kinase activity (Fig. 3a; and [11]). Indeed, in further support of this Src activation, we evaluated the phosphorylation of PLCγ on Tyr783, which is a Src substrate that is relevant for the signalling cascade initiated by GroPIns4*P*, in that it triggers the release of intracellular Ca^2+^ leading to actin remodelling [11]. In parallel with Src modifications, GroPIns4*P* treatment induced an increase in phosphorylation of PLCγ (Fig. 3a), thus confirming Src activation.

### GroPIns4*P*-induced Src activation in cells is mediated by Shp1

We next examined Src Tyr530 and PLCγ Tyr783 phosphorylations in NIH3T3 cells that over-expressed an inhibitory recombinant point-mutant of Shp1 (Shp1-C455S mutant), to establish whether Shp1 is required for GroPIns4*P*-induced Src activity. Mutation of the Cys residue to Ser at position 455 of the phosphatase domain results in a Shp1 catalytically inactive mutant that also functions as dominant-negative [37–39]. In NIH3T3 cells overexpressing Shp1 inactive mutant, GroPIns4*P* failed to induce Src dephosphorylation. In fact, the pTyr530-Src levels appeared to increase (Fig. 3a). GroPIns4*P* may induce the binding of this mutant to Src and protect the phosphorylated Tyr530 residue from the action of other phosphatases, causing increased phosphorylation of Tyr530. Under these conditions and in line with the hypothesis, no increase in the Src activating Tyr418 or PLCγ tyrosine phosphorylation was detected upon addition of GroPIns4*P* (Fig. 3a). Altogether, these observations indicate that GroPIns4*P*-induced Src activation requires and is mediated by Shp1 through dephosphorylation of Src at Tyr530.

We next examined whether GroPIns4*P* directly regulates Shp1 phosphatase activity against Src using isolated proteins. Phosphorylated Src was immunopurified from NIH3T3 cell lysates using a monoclonal antibody specific for the central portion of the protein, in order not to affect the phosphorylated C-terminal tail. Immunopurified Src was thus used as a substrate in in vitro phosphatase assays of recombinant Shp1 in the absence or presence of 50 μM GroPIns4*P*, and the phosphorylation of Src at Tyr530 was monitored by western blotting. As shown in Figs. 3b and c, GroPIns4*P* stimulated Shp1 activity and induced Src dephosphorylation, in line with the data obtained in intact cells (Fig. 3a).

### GroPIns4*P* promotes the association between Shp1 and Src in NIH3T3 cells

**Fig. 4:**
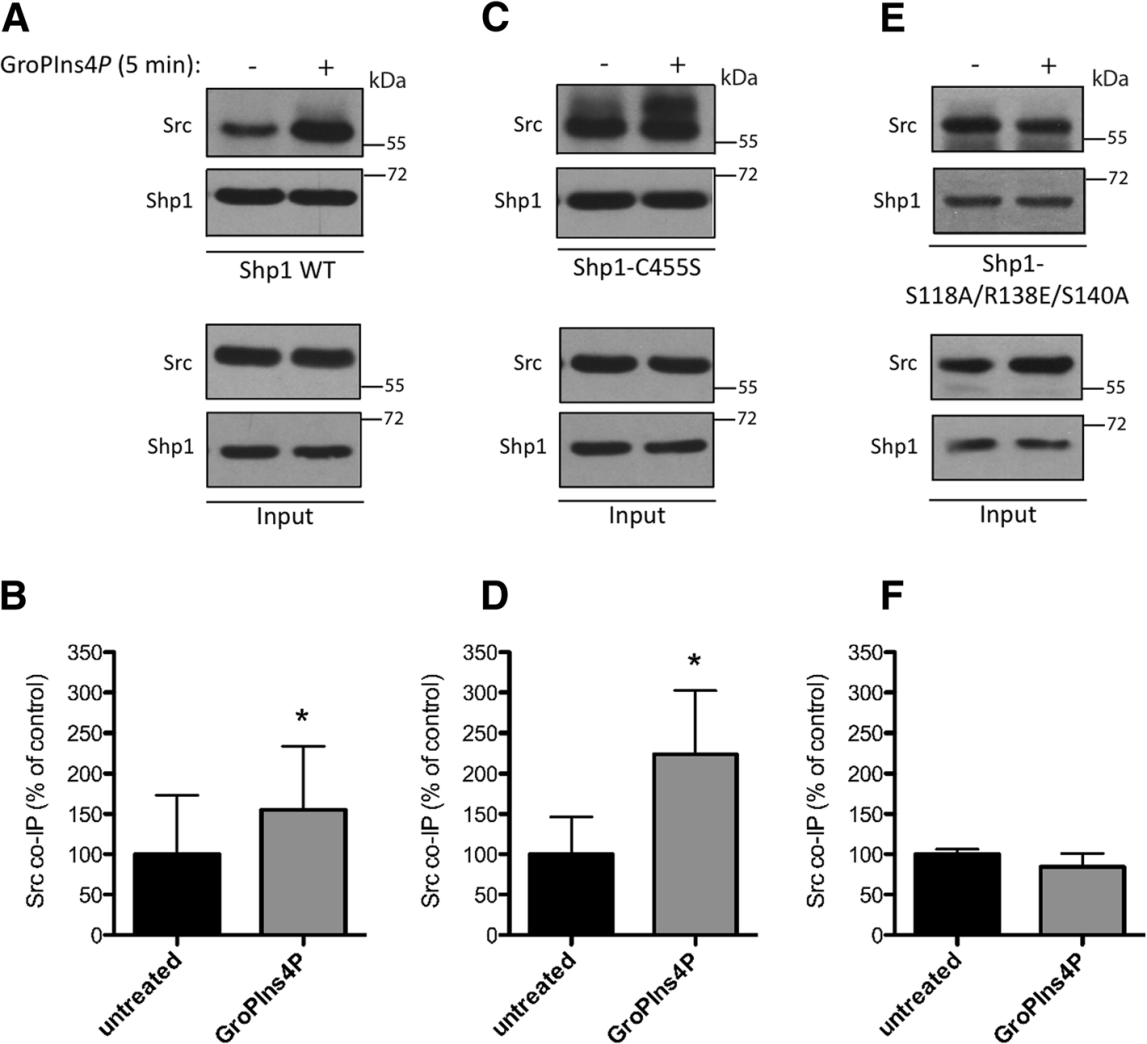
GroPIns4*P* favours the association between Shp1 and Src. **a, c, e** Interaction between Src and Shp1 wild type (**a**, Shp WT), Shp1-C455S (**c**, Shp1-C455S) or Shp1-S118A/R138E/S140A mutant (**e**, Shp1-S118A/R138E/S140A) was examined by immunoprecipitation (IP) with an anti-Shp1 antibody in serum-starved NIH3T3 cells over-expressing Shp1 WT, Shp1-C455S or Shp1-S118A/R138E/S140A mutant untreated (−) or treated (+) with 50 μM GroPIns4*P* for 5 min (as indicated). The expression levels of Shp1 WT, Shp1-C455S, Shp1-S118A/R138E/S140A and Src, examined in total lysates (input) indicate comparable amounts of proteins. Molecular weight standards (kDa) are indicated on the right of each panel. **b, d, f** Quantification of co-immunoprecipitated Src with Shp1 WT (**b**), Shp1-C455S (**d**) or Shp1-S118A/R138E/S140A mutant (**f**) using ImageJ analysis software. Data (GroPIns4*P*) are expressed as percentages of untreated sample (untreated) and as the means (±SD) of at least three independent experiments (Shp1 WT *n* = 4; Shp1-C455S *n* = 4; Shp1-S118A/R138E/S140A *n* = 3). *P < 0.05 (Student’s *t*-test)

Based on our results indicating that GroPIns4*P* binds to the cSH2 domain region of Shp1, we reasoned that the interaction between GroPIns4*P* and Shp1 might facilitate the association of Shp1 with, and ultimately dephosphorylation of, Src. To evaluate this possibility, we investigated whether Shp1 and Src physically associate with one another in a GroPIns4*P*-dependent manner, by immunoprecipitating lysates of NIH3T3 cells over-expressing Shp1 or a Shp1-C455S mutant with an anti-Shp1 antibody. Shp1 and Src co-immunoprecipitated as expected, and GroPIns4*P* increased their interaction by about 1.6-fold, compared with the control (Figs. 4a-b).

The binding of Shp1 with its substrates is known to be transient [40], and mutation of Cys455 of Shp1 within the catalytic pocket yields an enzyme in which the phosphatase activity is blocked and the affinity for the substrates is unaffected, thus maintaining binding [40]. Such mutants are called “substrate-trapping mutants” and have been used to identify in vivo substrates for several phosphatases. In cells over-expressing the Shp1-C455S mutant we observed that GroPIns4*P* caused a 2.2-fold increase in the interaction between Shp1-C455S and Src (Figs. 4c-d), an increase that was higher than that observed for Src binding to the Shp1 wild-type form (see Figs. 4a-b). These results are consistent with the concept that GroPIns4*P* causes an increase in the association of the substrate Src with its phosphatase Shp1 and that this association results in an increase in Src dephosphorylation and activity.

Next, to further define the molecular details of GroPIns4*P*-induced regulation of Shp1 activity, we evaluated whether the residues identified in the cSH2 domain that are crucial for the docking of GroPIns4*P* are also crucial for its biological activity. Therefore, we transfected NIH3T3 cells with Shp1 wild-type or Shp1 S118A/R138E/S140A mutant proteins, and analysed the association of Shp1 with Src under basal conditions or upon GroPIns4*P* treatment of NIH3T3 cells (as described above). We first observed that the binding of Src to the Shp1 S118A/R138E/S140A mutant was comparable to its binding with Shp1wt (Figs. 4e-f). Upon addition of GroPIns4*P*, no change in Shp1-Src complex formation was observed, indicating that the docking of GroPIns4*P* to the Ser118, Arg138 and Ser140 residues is required for the GroPIns4*P*-induced increase in the interaction of Shp1 with its substrate Src.

Taken together, our data indicate that GroPIns4*P* modulates the interaction between the phosphatase Shp1 and substrate Src and reveal three residues to be crucial for the binding of GroPIns4*P* to Shp1.

**Fig. 5:**
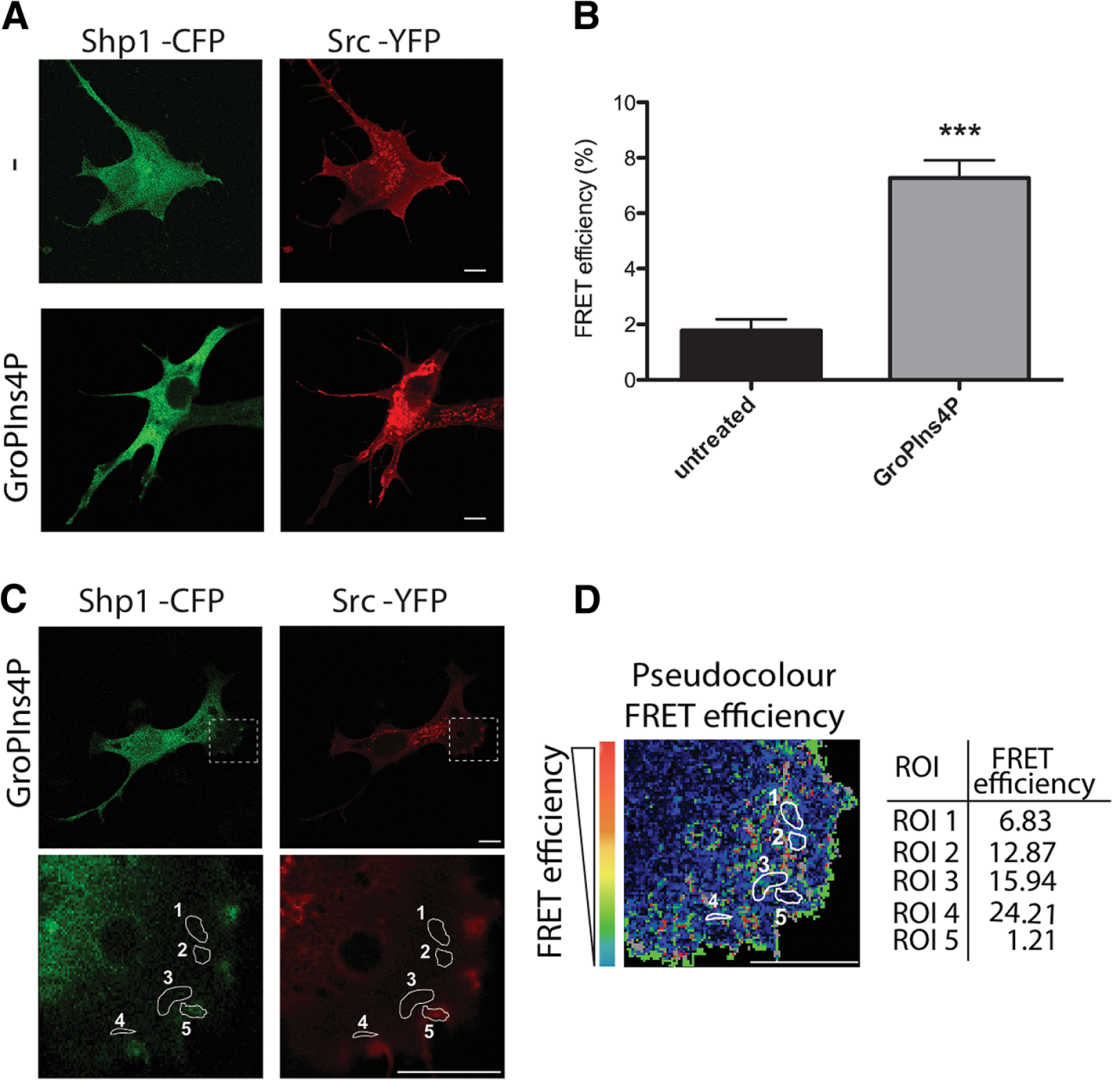
GroPIns4*P* promotes the association of Shp1 and Src within the lamellar region. **a** Representative confocal microscopy images of serum-starved NIH3T3 cells co-transfected with Shp1-CFP (green) and Src-YFP (red) for 24 h and then untreated (−) or treated with 50 μM GroPIns4*P* (GroPIns4*P*) for 5 min and subjected to Acceptor Photobleaching apFRET analysis. **b** Quantification of the FRET efficiency over the cells treated as in a. **c** Representative confocal microscopy images of serum-starved NIH3T3 cells co-transfected with Shp1-CFP (green) and Src-YFP (red) for 24 h and then treated with 50 μM GroPIns4*P* for 5 min before apFRET analysis. The dashed rectangle in the lamellar region (close to the plasma membrane) indicates where the FRET efficiency was analysed. **d** Representative colour-coded apFRET efficiency of the lamellar region (selected in a) where the colour-scale quantifies the degree of protein-protein interaction in each of the indicated regions of interest (ROIs 1 to 5, white circles; the FRET efficiency for each ROI is reported in the table on the right). Data are expressed as the means (±SD) (*n* = 15 cells/condition). ***P < 0.005 (Student’s *t*-test). Scale bars, 10 μm

Finally, to analyse the Shp1-Src interaction within intact cells, fluorescence resonance energy transfer (FRET) experiments were performed using a cyan fluorescent protein (CFP)-labelled construct of Shp1 (Shp1-CFP) and yellow fluorescent protein (YFP)-conjugated Src (Src-YFP). FRET signal was observed only when cells were exposed to GroPIns4*P* and not in untreated control cells (Figs. 5a-b). The statistical FRET signal analysis of Shp1-Src interaction under GroPIns4*P* treatment revealed an average efficiency of 9% (Fig. 5b). We further examined the distribution of this FRET over the GroPIns4*P*-treated cell and we showed that “hot spots” signals occur within the lamellar regions where the efficiency was over 20% (Figs. 5c-d).

Collectively, these data indicate that GroPIns4*P* activates Src in vitro by inducing Src dephosphorylation at Tyr530, and that this reaction is facilitated by an enhanced interaction between Shp1 and Src (Figs. 4 and 5).

### Shp1 is necessary for GroPIns4*P*-dependent actin ruffle formation and cell motility

Finally, we evaluated the involvement of Shp1 in the GroPIns4*P*-dependent induction of actin ruffles in NIH3T3 cells, which would be expected to occur based on the GroPIns4*P*-Src-actin ruffling cascade described here and in previous reports [10, 11]. To this end, we first used two well-characterised phosphatase inhibitors to reduce Shp1-phosphatase activity and then evaluated the effects on ruffle formation [41, 42].

**Fig. 6:**
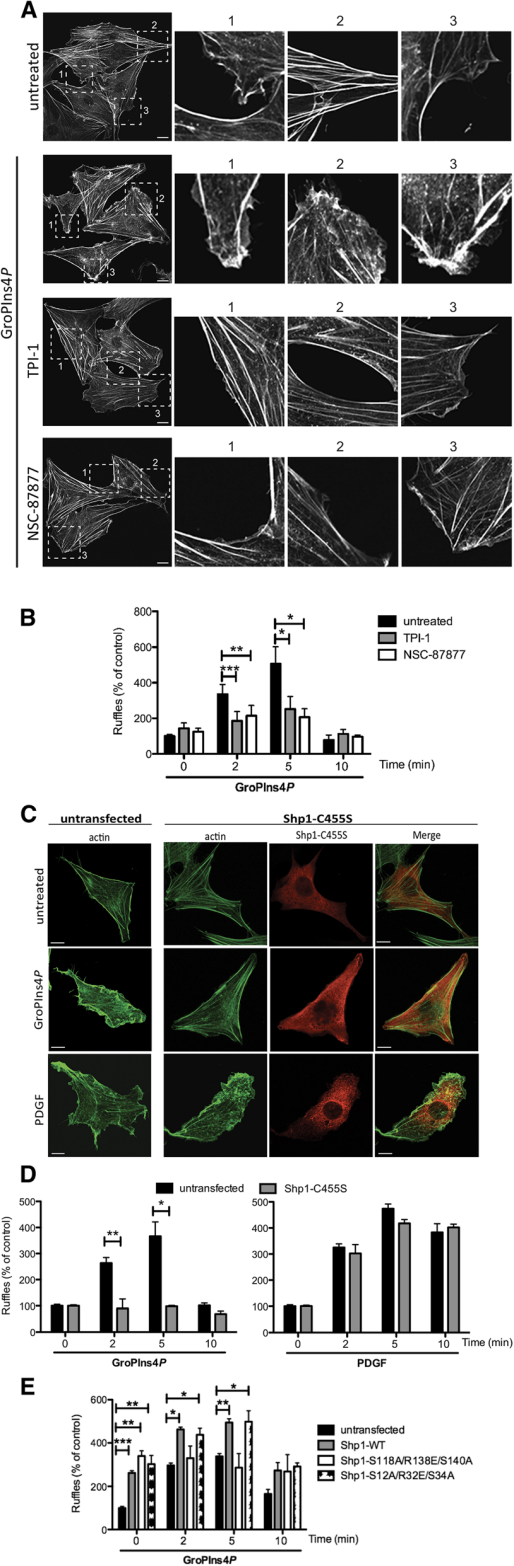
Shp1 directly mediates the GroPIns4*P*-induced actin ruffle formation. **a** Representative confocal microscopy images of serum-starved NIH3T3 cells untreated (untreated) or treated with 50 μM of GroPIns4*P* for 5 min alone (GroPIns4*P*) or in presence of the Shp1 inhibitors TPI-1 (TPI-1 + GroPIns4*P*) or NSC-87877 (NSC-87877 + GroPIns4*P*). The cells were fixed and processed for immunofluorescence analysis with FITC-labelled phalloidin. Zoom 1, 2 and 3: higher magnification images of the membrane area. **b** Quantification of actin ruffle formation (as percentage of untreated cells) of cells treated as in a (see the Methods). **c** Representative confocal microscopy images of NIH3T3 cells untransfected or transfected with the Shp1-C455S mutant for 8 h, starved for 24 h and then untreated (untreated) or treated with 50 μM GroPIns4*P* (GroPIns4*P*) or 10 ng/ml PDGF (PDGF) for 5 min. The cells were fixed and stained with an anti-Shp1 antibody and FITC-labelled phalloidin. **d** Quantification of actin ruffle formation (as the percentage of untreated cells) of cells treated as above. **e** Quantification of actin ruffle formation (as the percentage of untreated cells) of cells transfected with Shp1-WT, Shp1-S118A/R138E/S140A and Shp1-S12A/R32E/S34A mutants for 8 h, starved for 24 h and then treated with 50 μM GroPIns4*P* for the indicated times. Data are expressed as the means (±SD) of at least three independent experiments. ***P < 0.001; **P < 0.02; *P < 0.05 (Student’s *t*-test) calculated for each treatment versus untreated samples (untrasfected). Scale bars, 10 μm

A 2.5–3-fold increase in peripheral ruffles was clearly induced upon treatment with GroPIns4*P* (versus the control; Figs. 6a-b), which is comparable to our previous results [10, 11](see Methods for ruffle quantification). However, pre-treatment with either TPI-1 or NSC-87877 inhibitors strongly impair the ability of cells to assemble peripheral ruffles in response to GroPIns4*P*, suggesting the requirement of the Shp1 enzymatic activity to mediate localised GroPIns4*P* signalling. That said, the specificity of these inhibitors is limited, as they are known to target other phosphatases [42]. Thus, to specifically inhibit Shp1 activity, we over-expressed the dominant negative mutant Shp1-C455S [37, 38]. As expected, in the presence of the Shp1-C455S mutant, the addition of GroPIns4*P* did not lead to membrane ruffle formation, as previously observed with phosphatase/Shp1 chemical inhibitors (Figs. 6c-d). Thus, the tyrosine-phosphatase Shp1 is essential for GroPIns4*P*-dependent formation of membrane ruffles in NIH3T3 cells. The treatment with PDGF (10 ng/ml; used here as a positive control) produced an increase in ruffles of 3.5–4-fold the control that was not affected by Shp1-C455S mutant overexpression (Figs. 6c-d); this indicates that PDGF signalling in NIH3T3 cells diverges from that of GroPIns4*P*.

To further investigate the molecular basis of this GroPIns4*P*-induced effect on actin, we expressed the Shp1 S118A/R138E/S140A mutant, which cannot bind GroPIns4*P* in NIH3T3 cells and analysed peripheral ruffles upon GroPIns4*P* treatment. Over-expression of this mutant did not significantly affect membrane-ruffling formation compared with the wild-type protein, thus supporting the conclusion that these mutations do not directly impact Shp1 function (Fig. 6e). By contrast, the effect of GroPIns4*P* on ruffle formation was significantly inhibited, confirming that the Shp1 Ser118, Arg138 and Ser140 residues are crucial for GroPIns4*P*-mediated interaction between Shp1 and Src and the consequent induction of actin ruffles in NIH3T3 cells.

Based on the above results, we decided to investigate whether the second N-terminal SH2 domain (nSH2) is as crucial for the GroPIns4*P* activity as the cSH2 domain. We identified a sequence homologous to the S118/R138/S140 stretch of the cSH2 domain in the nSH2 domain of Shp1 at the positions S12/R32/S34. Mutation of these residues yielded a protein (Shp1 S12A/R32E/S34A mutant) that, when over-expressed in the NIH3T3 fibroblasts, responded to GroPIns4*P* and resulted in the induction of actin ruffles (Fig. 6e). These data indicate that the cSH2 domain of Shp1 is the crucial site initiating the cascade of the GroPIns4*P*-dependent actin ruffle formation. This conclusion is in line with the data obtained by different biophysical approaches pointing at a single GroPIns4*P* binding site on Shp1 (Additional file 1 Figure S1 and Figure S2).

### The GroPIns4*P*-increased cell motility and migration during wound healing involves Shp1

Membrane ruffles are often found at the leading edge of motile cells and are involved in cell spreading [43–45]. Hence, due to its ability to induce a local perturbation of the actin cytoskeleton, we analysed the role of GroPIns4*P* in cell motility by performing wound-healing assays on confluent monolayers of NIH3T3 cells.

**Fig. 7:**
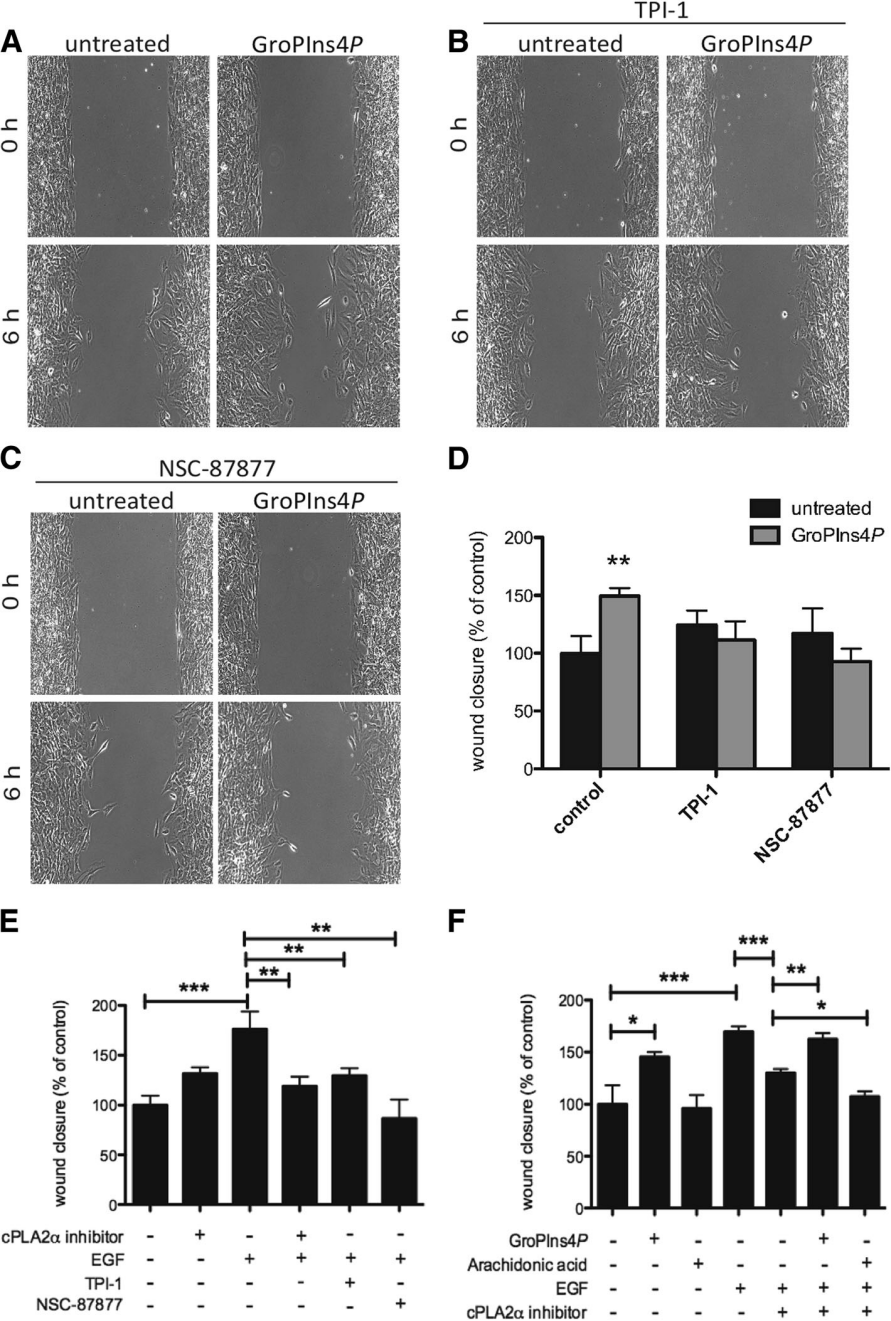
GroPIns4*P* binding to Shp1 facilitates and mediates EGF-induced wound closure in NIH3T3 cell monolayers. **a** Representative phase-contrast microscopy images after 6 h from scratching in serum-starved NIH3T3 cells untreated (untreated) or treated with 50 μM of GroPIns4*P* alone **(a)** or in presence of TPI-1 (**b**) or NSC-87877 Shp1 inhibitors (**c**), as indicated. **d** Quantification of wound healing as the percentage of the untreated control in cells treated as in a-c. **e, f** Cells without or with pre-incubation with the cPLA_2_α inhibitor, TPI-1, or NSC-87877 inhibitors (as indicated) were stimulated with 10 ng/ml EGF, 50 μM GroPIns4*P* or 10 μM arachidonic acid for 6 h. Quantifications of wound healing are presented as the percentage of the untreated controls. The differences between the untreated controls are not statistically significant. Data are expressed as the means (±SE) of at least three independent experiments. ***P < 0.001; **P < 0.02; *P < 0.05 (Student’s *t*-test) calculated for each treatment versus untreated samples (control). The cPLA_2_α inhibitor was used at 0.5 μM, but it was effective over a range of concentrations between 0.1 and 2 μM. Arachidonic acid was used at both 10 and 50 μM, with similar results

Cells exposed to 50 μM GroPIns4*P* showed a significantly improved wound-healing capability compared with the controls: GroPIns4*P*-treated cells started to migrate into the scratch area earlier than control cells, and wound closure was almost complete at 30 h. Quantifications performed at 6 h after scratching showed that wound closure was increased by 65% compared to the control (Figs. 7a, d), indicating a stimulatory role of GroPIns4*P* on fibroblast migration. Pre-treatment of NIH3T3 cells with either TPI-1 or NSC-87877 inhibitors (25 μM and 100 μM, respectively) completely abrogated GroPIns4*P*-stimulated wound closure (Figs. 7b-d), suggesting that Shp1 is required for GroPIns4*P*-induced cell motility in NIH3T3 cells. These data thus indicate that GroPIns4*P* in fibroblasts induces actin ruffles and cell motility and that Shp1 activity is necessary to mediate this GroPIns4*P* action.

### The EGF-receptor-dependent motogenic activity involves the stimulation of the cPLA_2_α-GroPIns4*P* pathway

Previous studies have indicated that glycerophosphoinositols can be produced by the hormonal activation of cPLA_2_α [2] and that specific increases in GroPIns4*P* levels are observed in NIH3T3 and Swiss3T3 fibroblasts upon exposure to epidermal growth factor (EGF) [3, 6]. Building on these and the current results, we carried out experiments to test the possibility that the GroPIns4*P*/Shp1 pathway might be involved in the EGF-induced cell migration.

NIH3T3 fibroblasts were serum-starved overnight and a wound-healing assay was conducted in the absence and presence of EGF (10 ng/ml). EGF stimulation induced a significant increase in cell migration; when cells were treated with Shp1 inhibitors this effect was reduced by 50% (with 25 μM TPI-1) and almost 100% (with 100 μM NSC-87877), indicating that Shp1 is required for EGF-induced cell motility.

To evaluate the role of the cPLA_2_α pathway and thus of GroPIns4*P* production in the motogenic effect of EGF, we repeated the above experiments in the presence of a specific cPLA_2_α inhibitor [46]. Pre-treatment of serum-starved NIH3T3 with 0.5 μM cPLA_2_α inhibitor strongly reduced EGF-stimulation of cell motility (Figs. 7e-f).

It has been reported that EGF-induced cytoskeleton organisation and cell motility also involve the activation of 5-lipoxygenase and formation of the arachidonic acid-derived leukotrienes [47, 48]. Considering that the inhibitors used in this study could also affect the formation of other metabolites of the arachidonic acid cascade, we directly investigated the role of arachidonic acid in the EGF-induced cell motility. In line with our previous report indicating that leukotriene E4 could induce stress fibres but not ruffle formation in fibroblasts [10], we observed that arachidonic acid (the leukotriene precursor; 10–50 μM) did not affect EGF-induced cell motility under the experimental conditions used in this study (Fig. 7f). When EGF-stimulated fibroblasts were treated as described above with the cPLA_2_α inhibitor, the lack of effect of the growth factor could be rescued by the addition of GroPIns4*P*, but not by the addition of arachidonic acid that instead, had a minor inhibitory effect when combined with the PLA_2_ inhibitor (this decrease was not further investigated since it was out of the scope of the present study; Fig. 7f). These data are consistent with our conclusion that the cPLA_2_α metabolite mediating the EGF-induced cell motility is mainly GroPIns4*P.*

Thus, the current and previous evidence collectively indicates that the motogenic activity initiated by EGF involves the cPLA_2_α/GroPIns4*P/*Shp1 pathway.

## Discussion

The main finding in this study is that the phosphatase Shp1 is an intracellular receptor of the phosphoinositide-derived cell mediator GroPIns4*P*. To our knowledge, this is the first receptor to be identified for one of these bioactive phosphoinositide metabolites. In addition, we present evidence that the GroPIns4*P*-controlled Shp1 activity is part of the signalling pathway activated by the EGF receptor to control cell motility. We propose that GroPIns4*P* endogenously-formed by EGF receptor-activated cPLA_2_α, interacts with Shp1, starting the Src-dependent signalling cascade that promotes ruffle formation and stimulation of cell motility [10, 11].

The definition of the Shp1 cSH2 domain as the specific GroPIns4*P* docking site represents a novel aspect of this study. Not all SH2 domains are recognised by GroPIns4*P*, as shown in the case of the Src SH2 domains where no direct binding of GroPIns4*P* could be observed [11]. This suggests that the specificity of the binding to Shp1, or other binding domains, can be due to specific structural and charge properties in the individual SH2 domains that affect their direct interaction with ligand such as GroPIns4*P,* or other phosphate moiety from proteins or lipids.

A SH2 domain ‘signature’ FLVR sequence characterises most SH2-domains, in which the highly conserved Arg is involved in the formation of an electrostatic interaction with the phosphate moiety of the ligands and thus plays a crucial role in phospho-Tyr binding [49]. Using SH2-mutants of Shp1, we show that the Arg residue at position 138 (in the conserved FLVR motif) is critical for GroPIns4*P* binding and for physiological action, since a lack of effect of GroPIns4*P* was observed in fibroblasts overexpressing the Shp1 S118A/R138E/S140A mutant. Considering that the SH2 domains play a role in the targeting of proteins to tyrosine-phosphorylated sites, thus allowing localised assembly and activation of signalling effectors, we propose that the binding of GroPIns4*P* to Shp1-SH2 domains is a means to recruit and compartmentalise Shp1 to a specific cell compartment, or to displace Shp1 when bound to membrane receptors/lipids, thus influencing the recognition of and binding to specific soluble protein substrates. Accordingly, we have shown that GroPIns4*P* enhances the association of Shp1 and Src in NIH3T3 fibroblasts within the lamella region next to the plasma membrane, but not at the plasma membrane (Fig. 5)*.*

The possibility to modulate Shp1 activity by GroPIns4*P* is of great pharmacological interest considering the diverse physiological and pathological conditions involving this phosphatase. Deletion of Shp1 is associated with a variety of pathologies including cancer and autoimmune diseases, such as allergic asthma, rheumatoid arthritis and multiple sclerosis [50]. Shp1 is a known negative regulator of cell cycle and of inflammatory cascades, including MAPKs, NF-KB and PI3K-Akt pathways [51]. Similarly, Shp1 directly targets the oncogenic Jak/STAT signalling pathway and thus contributes to apoptosis and tumor suppression in various cancer types, where an abolished or diminished Shp1 expression has been reported [52]. In this context, activating the Shp1 phosphatase activity has been demonstrated to be a promising therapeutic approach to induce apoptosis in cancer cells. Accordingly, the use of GroPIns4*P* may be a plausible approach to target this enzyme in cancer or in other appropriate contexts, with the aim to restore or stimulate its phosphatase activity.

An important notion proposed in this study is that the action of GroPIns4*P* on the actin cytoskeleton may mediate the EGF effects on cell migration. EGF is known to promote PLA_2_ activation [53–55] through several independent signalling pathways [56], that may thus lead to GroPIns4*P* [6] and arachidonic acid formation. This is the case for the MEK-dependent phosphorylation cascade that has been involved in both the activation of PLA_2_ [57] and formation of GroPIns4*P* [6]. Similarly, the activation of PLCγ and cytosolic Ca^2+^ increase lead to PLA_2_ activation [58]. Within these diverse signalling pathways the GroPIns4*P* formed by EGF-dependent stimulation [3, 6] may represent the resulting common messenger mediating the EGF-dependent activation of cell motility.

Indeed, here we demonstrate that the EGF-dependent stimulation of cell motility is inhibited under conditions that reduce the formation of GroPIns4*P*. Other mediators are certainly involved in the EGF action; however, the virtually overimposable effects on Rac activation and ruffle formation produced by both EGF [59–61] and exogenously-added GroPIns4*P* [11] suggest that the endogenous formation of GroPIns4*P* plays a significant role in the motogenic component of the EGF signalling. In this scenario, GroPIns4*P* could cooperate with other EGF-regulated pathways to coordinate actin remodelling and cell migration.

Another evidence linking EGF-induced cPLA_2_α activation and GroPIns4*P* production is related to their cell localisation. EGF is known to induce the translocation of cPLA_2_α from the cytosol to the plasma membrane [53, 55]. In NIH3T3 cells pre-treated with a cPLA_2_α inhibitor, cPLA_2_α translocation in response to EGF stimulation was partially blocked; thus, cPLA_2_α remained primarily cytosolic (Additional file 1 Figure S4). The lack of localisation of cPLA_2_α at the leading edge of fibroblasts could prevent the local production of GroPIns4*P*, thus negatively affecting actin dynamics and cell movement (Fig. 7). In this way both the catalytic activity and localisation of cPLA_2_α would result relevant for the EGF-dependent cell motility mediated by GroPIns4*P.*

Arachidonic acid, the other likely product of PLA_2_ activation, does not appear to affect cell motility under the experimental conditions reported, however it has been shown to promote the transcription of genes relevant in cell motility (at concentrations between 5 and 15 μM) [62, 63]. Thus PLA_2_ metabolites might control motility directly by acting on the Shp1/Src pathway through GroPIns4*P*, and at the transcriptional level through arachidonic acid. Finally, and as previously reported [10], GroPIns4*P* cannot exert its effect upstream of either PI3K or PLA_2_, enzymes that are also involved in the EGF-induced ruffle formation: their inhibition did not affect the ability of GroPIns4*P* to induce membrane ruffles, further supporting the relevance of the GroPIns4*P*/Shp1/Src pathway.

At the same time, it should be considered that Src can also be activated by direct binding to the EGF-receptor initiating diverse signalling cascades involving kinases such as MAP, P38 and PI3, that in turn lead to the remodelling of focal adhesions and cell migration through the phosphorylation of specific downstream substrates [64, 65]. These represent pathways that may be active in parallel or in alternative to the PLA_2_/GroPIns4*P*/Shp1-dependent Src activation reported in this study and that may represent either a redundancy of signals or, most probably, a response to diverse cell conditions.

In addition to EGF, other growth factors and ligands, such as insulin, lysophosphatidic acid or ATP, modulate the endogenous GroPIns4*P* level by activating cPLA_2_α [6], while the level of GroPIns are modulated, for example, by the Fc receptor-dependent stimulation of cPLA_2_α in macrophages [7], or the *Ret/PTC* oncogene-dependent PLA_2_ activation in human thyroid cancer cells [66]. The formation of the glycerophosphoinositols in normal and pathological conditions might thus represent a widespread mechanism of regulation of signalling pathways leading to cell motility. GroPIns4*P* is also likely to play an important role in other cell responses such as the immuno-inflammatory response [9], depending on the cell type involved or on specific physiopathological conditions. With the elucidation of the interaction of Shp1 with GroPIns4*P* all these previous studies can be re-evaluated in the light of the known cellular roles of this specific receptor. Similarly, and possibly more importantly, the numerous pathological conditions that have been related to the inhibition of Shp1, could now be directly modulated by the use of the exogenous administration of GroPIns4*P* as reported in this study. GroPIns4*P* represents an ideal lead compound for developing therapeutic approaches requiring the activation of Shp1. The present findings therefore add to the concept that GroPIns4*P* and GroPIns can be pharmacologically exploited as natural, non-toxic compounds of potential use in proliferative and immuno-inflammatory diseases (patent US9351983 B2 “Use of glycerophosphoinositols for the treatment of septic shock”).

## Conclusions

**Fig. 8:**
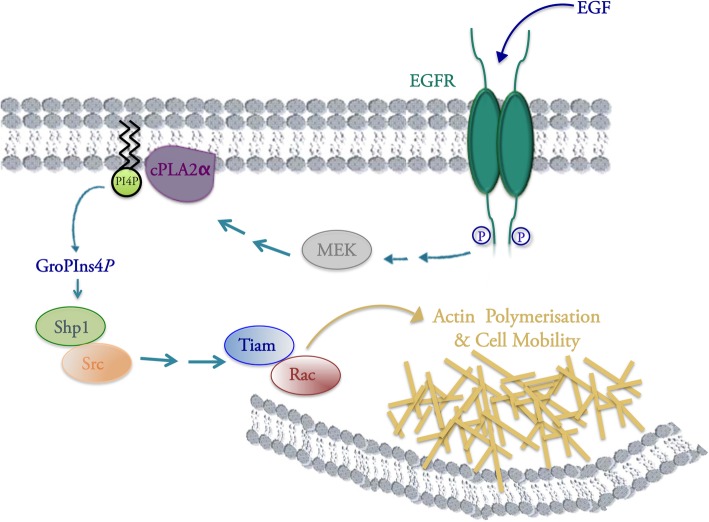
Schematic representation of the signalling cascade involved in the formation of GroPIns4*P* in NIH3T3 cells. In NIH3T3 cells, activation of the EGF receptor (EGFR) can lead to activation of cPLA_2_α through the involvement of MEK kinase. cPLA_2_α activation results in hydrolysis of PtdIns4*P* and release of GroPIns4*P*, which binds to Shp1 and promotes its association with Src. Once activated Src triggers a signalling cascade culminating with the formation of Tiam/Rac complex at the plasma membrane and consequent formation membrane ruffles with stimulation of cell motility. See text for details

With this study we show that the phosphatase Shp1 is the first-identified cellular receptor of the bioactive metabolite GroPIns4*P*, and delineate a signalling pathway involved in the regulation of cell motility that is initiated by the activation of the EGF-receptor and followed by the activation of cPLA_2_α, GroPIns4*P* formation and Shp1-direct interaction with, and activation of, Src. This in turn initiates a phosphorylation cascade leading to actin polymerisation, hence cell motility (Fig. 8). These data point also at the pharmacological use of GroPIns4*P* as a new tool to modulate Shp1 and Src activity, with several implications for various physiological and pathological conditions.

## Additional file

Additional file 1: **Figure S1.** Direct binding of GroPIns4P to Shp1. **Figure S2.** Binding of GroPIns4P to the Shp1 WT and S118A/R138E/S140A mutant evaluated by isothermal titration calorimetry (ITC). **Figure S3.** Binding of GroPIns4P to the Shp1 S118A/R138E/S140A mutant. **Figure S4.** Localisation of endogenous cPLA2α in NIH3T3 cells. **Table S1.** Acquisition parameters for NMR experiments performed on the cSH2 domain of Shp1. **Table S2.** Docking calculations. **Table S3.** List of proteins identified from proteomic analysis. (DOCX 2706 kb)

## Abbreviations

CaMKII: Calcium-calmodulin kinase II
cPLA_2_: Cytosolic-phospholipase A_2_
EGF: Epidermal growth factor
FRET: Fluorescence resonance energy transfer
GEF: GTP exchange factor
GroPIns4*P*: Glycerophosphoinositol 4-phosphate
Ins4*P*: Inositol 4-phosphate
NMR: Nuclear magnetic resonance
PDGF: Platelet-derived growth factor
SH2: Src-homology 2
SPR: Surface plasmon resonance
