# Supplementary material for: A signalling cascade involving receptor-activated phospholipase A2, glycerophosphoinositol 4-phosphate, Shp1 and Src in the activation of cell motility
Source: Cell Commun Signal. 2019 Mar 1;17:20. doi: 10.1186/s12964-019-0329-3 (PMC6396489; doi:10.1186/s12964-019-0329-3)
Supplement: Supplementary file 1 — Figure S1. Direct binding of GroPIns4P to Shp1. Figure S2. Binding of GroPIns4P to the Shp1 WT and S118A/R138E/S140A mutant evaluated by isothermal titration calorimetry (ITC). Figure S3. Binding of GroPIns4P to the Shp1 S118A/R138E/S140A mutant. Figure S4. Localisation of endogenous cPLA2α in NIH3T3 cells. Table S1. Acquisition parameters for NMR experiments performed on the cSH2 domain of Shp1. Table S2. Docking calculations. Table S3. List of proteins identified from proteomic analysis. (DOCX 2706 kb) [file 12964_2019_329_MOESM1_ESM.docx]

**Varone *et al*, 2019 Electronic supplementary material**

**Fig. S1** Direct binding of GroPIns4*P* to Shp1. Related to Fig. 1. **a** Dose-response effect of GroPIns4*P* on the Shp1-Trp fluorescence emission (ΔF) at 332 nm. GroPIns4*P* concentrations are indicated on the right. **b** Binding of Shp1 on a GroPIns4*P*-Bio-functionalised chip (sensorgram) analysed by surface plasmon resonance (SPR), as a function of time. Shp1 concentrations are indicated under the panel.

**Fig. S2** Binding of GroPIns4*P* to the Shp1 WT and S118A/R138E/S140A mutant evaluated by isothermal titration calorimetry (ITC). Related to Fig. 2. **a** ITC experimental data for the titration of (a) Shp1 WT (K_D_ 0.3 μM) and (b) Shp1 S118A/R138E/S140A mutant with GroPIns4*P.* In the latter case the K_D_ was about one order of magnitude higher that in the former however, due to the very small heat effect registered (b), the value obtained were not with sufficient confidence; (c) heat of dilution of GroPIns4*P* in the buffer. **b** Binding isotherms for the titration of Shp1 WT (squares) and Shp1 S118A/R138E/S140A mutant (triangles) with GroPIns4*P*. Similar experiments performed in the presence of Ins4*P* (50 μM) did not indicate any specific binding of this compound to Shp1.

**Fig. S3** Binding of GroPIns4*P* to the Shp1 S118A/R138E/S140A mutant. Related to Fig 2. Dose-response effect of GroPIns4*P* on Shp1 S118A/R138E/S140A mutant-Trp fluorescence emission (ΔF) at 332 nm. GroPIns4*P* concentrations are indicated on the right.

**Fig. S4** Localisation of endogenous cPLA_2_α in NIH3T3 cells. Related to Fig 7. Fibroblasts were serum-starved for 24 h and subsequently incubated for 5 min with 10 ng/ml EGF in the absence and presence of a 15-min pre-treatment with 0.5 μM cPLA_2_α inhibitor. The cells were fixed and stained with the anti-cPLA_2_α antibody. Scale bars, 10 µm.

**Table S1. Acquisition parameters for NMR experiments performed on the cSH2 domain of Shp1.** Related to Figure 2.

| Experiments^a^ | Dimension of acquired data  (nucleus) | | | Spectral width (ppm) | | | n^b^ |
| --- | --- | --- | --- | --- | --- | --- | --- |
|  | t1 | t2 | t3 | F1 | F2 | F3 |  |
| ^1^H-^15^N-HSQC | 256(^15^N) | 1024(^1^H) |  |  |  |  | 8 |
| HNCACB | 128(^13^C) | 48 (^15^N) | 2048(^1^H) | 76 | 38 | 16 | 16 |
| CBCA(CO)NH | 128(^13^C) | 48 (^15^N) | 2048(^1^H) | 76 | 38 | 16 | 16 |
| HNCO | 88 (^13^C) | 48 (^15^N) | 2048(^1^H) | 16 | 38 | 16 | 16 |
| HN(CA)CO | 88 (^13^C) | 48 (^15^N) | 2048(^1^H) | 16 | 38 | 16 | 16 |
| HNCA | 96 (^13^C) | 48 (^15^N) | 2048(^1^H) | 38 | 38 | 16 | 16 |
| HN(CO)CA | 96 (^13^C) | 48 (^15^N) | 2048(^1^H) | 38 | 38 | 16 | 16 |
| ^15^N-edited [^1^H-^1^ H]-NOESY^c^ | 192 (^1^H) | 56 (^15^N) | 2048(^1^H) | 16 | 44 | 16 | 16 |

^a^ Experiments were carried out on a 700 MHz Bruker spectrometer equipped with a triple resonance cryoprobe at 298 K. All of the triple resonance (TCI 5mm) probes used were equipped with Pulsed Field Gradients along the z-axis. ^b^ number of acquired scans ^c^15N-edited 3D NOESY-HSQC experiments were acquired with a mixing time value of 100 ms.

**Table S2. Docking calculations.** Related to Figure 2.

|  | **Cluster 1^a^** | **Cluster 2** | **Cluster 3** | **Cluster 4** | **Cluster 5** |
| --- | --- | --- | --- | --- | --- |
| **HADDOCK Score ^b^** | -72.4  (3.4)   \|  \| \| --- \| \|  \| \|  \| \|  \| | -71.1  (1.7) | -68.8  (3.5) | -67.6  (4.6) | -66.1  (0.4) |
| **RMSD (Å) ^c^** | 0.3  (0.2) | 0.3  (0,2) | 0.4  (0.2) | 0.3  (0.2) | 0.3  (0.2) |
| **Number of structures** | 97 | 40 | 12 | 12 | 79 |
| **BSA (Å^2^) ^d^** | 342.8 (24.2) | 555.9 (14.8) | 455.8 (15.1) | 486.9 (13.1) | 480.8 (23.1) |
| **Einter ^e^** | -393.2 (23.4) | -281.6 (15.5) | -370.9 (26.7) | -339.7 (14.1) | -319.7 (20.2) |
| **Enb ^f^** | -393.7 (23.6) | -281.7 (15.5) | -371.1 (26.7) | -339.9 (14.3) | -319.9 (20.2) |

Statistics on the five clusters of the structural models of the cSH2 domain-GroPIns4*P* complex obtained through HADDOCK2.2 calculations. Averages (with their standard deviations reported in parenthesis) were calculated over the best four model structures.

^a^ Cluster rank according to the HADDOCK score. ^b^ The HADDOCK score is defined as the weighted sum of different energetic terms, such as: van der Waals energy, electrostatic energy, distance restraint energy, buried surface area, binding energy and desolvation energy. ^c^ Backbone root-mean-square deviation (RMSD) from the lowest HADDOCK score structure in each cluster. Some individual energy terms are also reported: ^d^ Buried surface area, ^e^ binding energy, ^f^ non-bonded interaction energy.

**Table S3. List of proteins identified from proteomic analysis.**

| **Swiss-Prot Code** | **Protein name** | **Mascot Score** |
| --- | --- | --- |
| Q9JKR6 | Hypoxia up-regulated protein 1 | 155 |
| P42567 | Epidermal growth factor receptor substrate 15 | 120 |
| P70248 | Myosin-If | 76 |
| Q64727 | Vinculin | 169 |
| Q8K4Z5 | Splicing Factor 3A subunit 1 | 75 |
| Q9EQK5 | Major vault protein | 179 |
| Q61699 | Heat shock protein 105 kDa | 110 |
| Q6P5F9 | Exportin-1 | 96 |
| Q11011 | Puromycin-sensitive aminopeptidase | 68 |
| P29351 | Tyrosine-protein phosphatase non-receptor type 6 (Shp1) | 189 |
| Q8BMF4 | Dihydrolipoamide acetyltransferase PDH-E2 | 170 |
| Q922R8 | Protein disulfide-isomerase A6 | 402 |
| P47738 | Aldehyde dehydrogenase | 314 |
| Q62465 | Synaptic vesicle membrane protein VAT-1 | 219 |
| Q99K87 | Serine hydroxymethyltransferase | 218 |
| P80314 | T-complex protein 1 subunit beta | 218 |
| Q61598 | Rab GDP dissociation inhibitor beta | 193 |
| Q9CZ13 | Cytochrome b-c1 complex subunit 1 | 103 |
| Q9CZU6 | Citrate synthase | 139 |
| Q9CR16 | 40 kDa peptidyl-prolyl cis-trans isomerase | 114 |
| Q9CQV8 | 14-3-3 protein beta/alpha | 215 |
| P34022 | Ran-specific GTPase-activating protein | 120 |
| O70435 | Proteasome subunit alpha type-3 | 118 |
| P42125 | 3,2-trans-enoyl-CoA isomerase | 104 |
| Q99PT1 | Rho GDP-dissociation inhibitor 1 | 207 |
| P47963 | 60S ribosomal protein L13 | 182 |
| Q61599 | Rho GDP-dissociation inhibitor 2 | 167 |
| P54116 | Erythrocyte band 7 integral membrane protein | 94 |
| P00375 | Dihydrofolate reductase | 76 |
| P62751 | 60S ribosomal protein L23a | 91 |
